# Joint-based description of protein structure: its application to the geometric characterization of membrane proteins

**DOI:** 10.1038/s41598-017-01011-z

**Published:** 2017-04-21

**Authors:** Jayaraman Thangappan, Sangwook Wu, Sun-Gu Lee

**Affiliations:** 1 Department of Chemical Engineering, Pusan National University, Busan, 609-735 Republic of Korea; 2 Department of Physics, Pukyong National University, Busan, 608-737 Republic of Korea

## Abstract

A macroscopic description of a protein structure allows an understanding of the protein conformations in a more simplistic manner. Here, a new macroscopic approach that utilizes the joints of the protein secondary structures as a basic descriptor for the protein structure is proposed and applied to study the arrangement of secondary structures in helical membrane proteins. Two types of dihedral angle, Ω and λ, were defined based on the joint points of the transmembrane (TM) helices and loops, and employed to analyze 103 non-homologous membrane proteins with 3 to 14 TM helices. The Ω-λ plot, which is a distribution plot of the dihedral angles of the joint points, identified the allowed and disallowed regions of helical arrangement. Analyses of consecutive dihedral angle patterns indicated that there are preferred patterns in the helical alignment and extension of TM proteins, and helical extension pattern in TM proteins is varied as the size of TM proteins increases. Finally, we could identify some symmetric protein pairs in TM proteins under the joint-based coordinate and 3-dimensional coordinates. The joint-based approach is expected to help better understand and model the overall conformational features of complicated large-scale proteins, such as membrane proteins.

## Introduction

Protein structures are strongly related to their physical properties, such as folding, stability, and function. They also include information on how proteins have evolved and connected with each other. A study of the structural and conformational features of proteins is one of the most significant issues in protein science. Traditionally, many studies have examined protein structures with an all atom-based description^1, 2^. The Ramachandran’s plot^1^ with the backbone dihedral angle ϕ (N-C_α_) and ψ (C_α_-C) is a representative way of microscopic descriptions of the protein structure. The Ramachandran plot shows the allowed and disallowed values of the dihedral angles of amino-acid residues of polypeptide backbone chain. The plot provides an understanding of the local and global features of protein structures. For example, the Ramachandran plot is built in the PROCHECK^3^ and WHAT_CHECK^4^ to verify the stereochemical quality of the protein structure. Recently, it has been used widely to validate the protein structure generated from homology modeling^5^ within the frame of molecular dynamics simulations.

**Figure 1:**
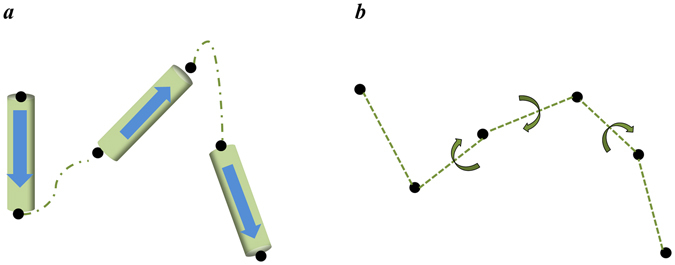
Schematic of the joint-based approach for macroscopic description of the protein structure. (**a**) Secondary structure-based description of the protein structure. The arrow means the sequence of amino acids of the protein with increasing residue numbers. (**b**) Newly devised joint-based description of the protein structure using the joints of secondary structures and their dihedral angles.

The protein geometry has also been studied at the coarse-grained level: C_α_ atom-based coordinates^6–9^, residue-based coordinates^10, 11^ and secondary structure-based coordinates^12–14^. The coarse-grained models for the protein structure enable an understanding of the protein conformations in more simplistic manner, which may have an advantage in studying large-scale proteins, such as multi-protein complex or membrane proteins with several transmembrane (TM) helices. This paper proposes a new macroscopic description method for the protein structures. In general, protein structures can be described using the secondary structures such as α helices, β sheets and loops as the basic units at the macroscopic level (Fig. 1a). Our new strategy is to use the joints of secondary structures as the basic constituents for a description of the protein structure and to study the protein conformational features by examining the 3-dimensional arrangement of the joints with their dihedral angles (Fig. 1b).

Here, the macroscopic description method is applied to study the conformational features of the membrane proteins. Membrane proteins have a range of cellular functions, such as signal transduction (protein kinase)^15, 16^, ion channeling (potassium channel)^17–19^, energy metabolism (voltage-dependent anion channel)^20^, and drug recognition (multidrug resistance protein)^21, 22^. Most membrane proteins are composed of transmembrane (TM) helices. Various cellular functions of the membrane proteins are quite relevant to their diverse conformations inside the lipid membrane. The complexity of the membrane protein can be understood within the frame of the statistical distribution of the conformations of the TM helices of the membrane proteins^23^. In this study, the conformation of the TM helices was investigated by the new description method using the structural joints at the macroscopic level. The non-homologous structures of the membrane proteins from Protein Data Bank were selected and analyzed using the joint-based method. Some common and interesting features of membrane proteins reflecting the conformational heterogeneity and specificity are suggested based on an analysis of the conformations of non-homologous membrane proteins with the dihedral angles of the joints.

## Results

### Macroscopic description of membrane protein structure using joint-based approach

**Figure 2:**
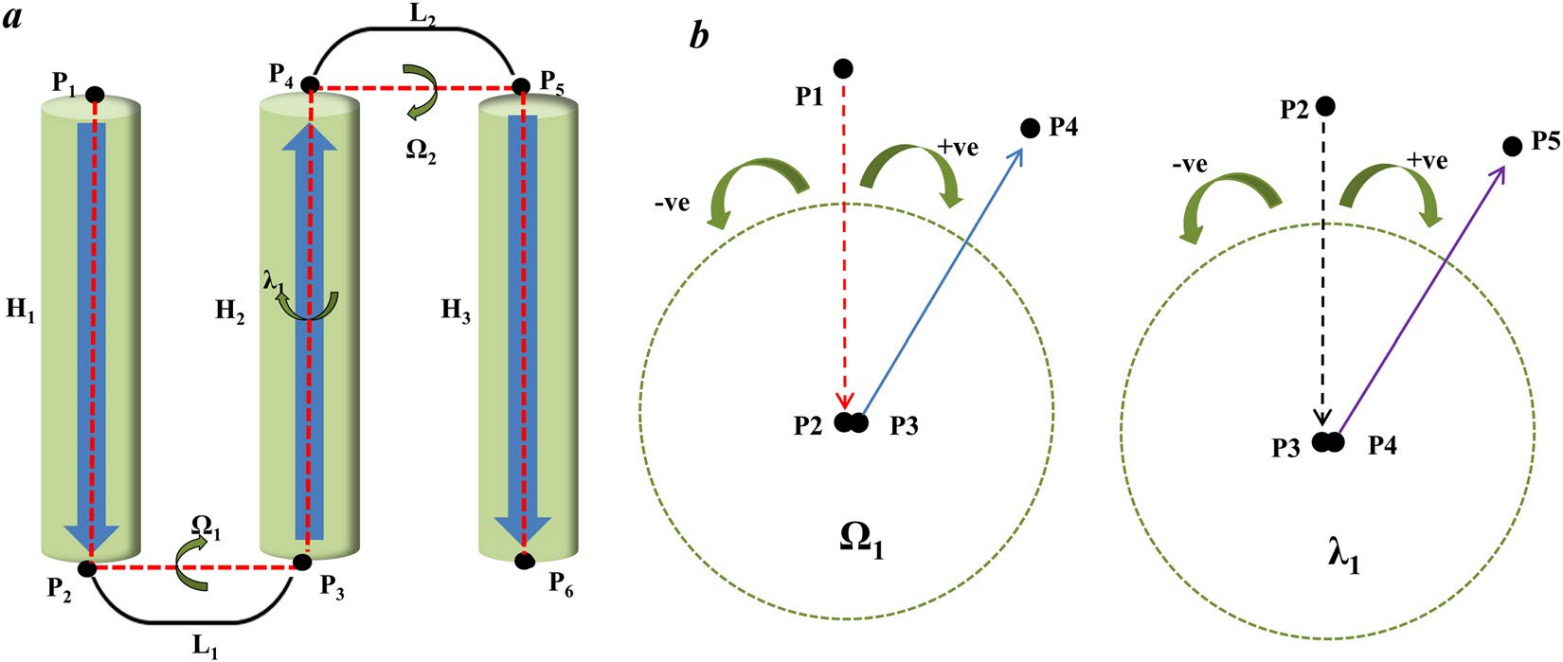
Joint-based description of membrane proteins with three helices and two loops. (**a**) Assignment of the Ω type and λ type dihedral angles. H_1_ to H_3_ are helices, L_1_ to L_2_ are loops, and P_1_ to P_6_ are joint points. Ω-type dihedral angles, such as Ω_1_, are defined by the four joint points in the Helix-Loop-Helix, such as P_1_, P_2_, P_3_, and P_4_. The λ-type dihedral angles, such as λ_1_, are defined by the four joint points in the Loop-Helix-Loop, such as P_2_, P_3_, P_4_ and P_5_. (**b**) Assignment of the positive and negative signs for dihedral angles. The positive (+) sign and negative (−) signs represent the clockwise and counter-clockwise angles, respectively, in the projections for the dihedral angles. The figures present the projections for Ω_1_ and λ_1_.

Most membrane proteins with TM helices display a repetition of the TM helix and loop, as shown in Fig. 2a. To present the structure based on the joint approach, a set of joints associating the helices and loops were selected. In particular, the C-alpha carbon of the beginning and ending residues of each TM helix were considered as structural joining points, and employed as structural elements of the protein structure. The spatial arrangement of the joint points was determined by the dihedral angles between the two joint points. For example, 6 joints (P_1_, P_2_, P_3_, P_4_, P_5_ and P_6_) can be assigned for a protein composed of three helices (H_1_, H_2_ and H_3_) and two loops (L_1_ and L_2_) (Fig. 2a). The first dihedral angle involving four joints (P_1_, P_2_, P_3_ and P_4_) can be ascertained by measuring the angle between two planes made by (P_1_, P_2_, P_3_) and (P_2_, P_3_, P_4_). Similarly, the second dihedral angle can be found by applying the structural points (P_2_, P_3_, P_4_, P_5_), and the (P_3_, P_4_, P_5_ and P_6_) joints are used to determine the third, and so on. The dihedral angles are classified into two types: Ω and λ types. The first and third dihedral angles determined by the four joints in the Helix-Loop-Helix correspond to type Ω; they are denoted as Ω_1_ and Ω_2_, respectively. In a similar way, the dihedral angles determined by the four joints in a Loop-Helix-Loop, such as the second dihedral angle, correspond to the type λ, denoted as λ_1_. The conformation of TM helices of the membrane proteins can be represented by a set of two types of dihedral angles (Ω_1_, λ_1_, Ω_2_, λ_2_, Ω_3_ …) composed of a set of joints (P_1_, P_2_, P_3_, P_4_ …) at the macroscopic level. For the dihedral angles, the clockwise angle (from 0 to 180 degrees) was assigned as a positive value and counter-clockwise angle as a negative value (Fig. 2b). The algorithm to define the structural joints and the dihedral angles between the joint points is shown in detail (**See** ***Methods***).

### Dataset of target membrane proteins

A total of 103 non-homologous membrane proteins with 3 to 14 transmembrane (TM) helices were used as a dataset (Table 1). The dataset was obtained from the protein data bank (PDB) by characterizing all the resolved polytopic membrane spanning structures. The ***Methods*** section presents a detailed procedure to obtain the dataset. Briefly, (i) 2600 membrane proteins with X-ray crystal structures were collected from PDB, (ii) 959 proteins with only α helices were selected from them, and (iii) finally 103 non-homologous monomeric chains with 3 TM to 14 TM helices were selected from the 959 proteins. To validate how much the selected non-homologous protein dataset is complete, a structural homology detection study was performed using the 103 non-homologous proteins against whole 959 helical TM proteins, similarly to the previous study^24^. The selected 103 proteins could cover around 90 to 97% of the 959 protein structures depending on the RMSD threshold range (3 to 5 Å) for structural homology. This suggests that the selected dataset represents the whole dataset quite completely. The target dataset of the 103 protein structures were analyzed using the joint-based approach. Table S1 in Supplementary Information presents the Ω and λ type dihedral angles for the 103 non-homologous membrane proteins.

**Table 1:** Selected non-homologous helical membrane protein structures, their PDB IDs, and number of Ω type and λ type dihedral angles used in this study.

Group^a^	PDB IDs	# of Structures	# of Ω types^b^	# of λ types^c^
*3 TM*	*2ZT9B*, *2BHWA*, *3ZE5A*, *4O9PA*, *1YQ3C*, *4X5MA*, *3RKOA*, *5AJIA & 4U1WA*	*9*	*18*	*9*
*4 TM*	*4HKRA*, *2BL2A*, *4YMKA*, *2UUHA*, *5TCXA*, *5ER7A*, *4WD8A*, *1KQFC*, *4RI2A*, *1Q90B*, *3EAMA*, *5DIRA & 2ZUQA*	*13*	*39*	*26*
*5 TM*	*4UC1A*, *3TUIA*, *4A2NB*, *1Q16C*, *3WU2A*, *3WVFA*, *3RGBC*, *4U9NA & 4NV5A*	*9*	*36*	*27*
*6TM*	*3RGBB*, *4MRSA*, *3UX4A*, *4B4AA*, *3H90A*, *5JWYA*, *5I32A*, *4P6VE*, *3RVYA*, *4O6MA*, *2XOWA*, *4XU4A*, *1OKCA*, *3RLBA*, *4O6YA*, *3WU2B*, *3B4RB &2R9RB*	*18*	*90*	*72*
*7 TM*	*2Z73A*, *5SYTA*, *4PGRA*, *2DYRC*, *5CTGA*, *5AZBA & 5EGIA*	*7*	*42*	*35*
*8 TM*	*5DWYA*, *2VPZC*, *4QTNA*, *4J7CI*, *3RFUA*, *4P02A &3TIJA*	*7*	*49*	*42*
*9 TM*	*4O9PB*, *4TQ4 & 4Q2GA*	*3*	*24*	*21*
*10TM*	*2ZXEA*, *3QNQA*, *4P6VB*, *4QUVA*, *5I20A*, *2NQ2A*, *3V5UA*, *4WISA*, *3M73A*, *4N7WA*, *1RH5A*, *3K3FA*, *3QKYA*, *4J72A*, *1OTSA & 4WGVA*	*16*	*144*	*128*
*11 TM*	*4RP9A*, *4R0CA*, *3B9YA*, *4K1CA & 1JB0A*	*5*	*50*	*45*
*12 TM*	*4GC0A*, *3GIAA*, *3K07A*, *5DQQA*, *4KPPA*, *4ATVA*, *4LZ6A*, *5KO2A*, *2DYRA & 4C7RA*	*10*	*110*	*100*
*13 TM*	*3RCEA*, *3S8GA*, *4CZ8A & 4F35A*	*4*	*48*	*44*
*14 TM*	*3QE7A &4IKVA*	*2*	*26*	*24*
*Total numbers*		*103*	*676*	*573*

All protein names and their classifications used in this work are described in the Supplementary Information Table S5.

^a^Group was categorized according to the TM helical numbers in the proteins.

^b^Total number of Ω type angles in the group.

^c^Total number of λ type angles in the group.

In this study, we measured the dihedral angles of joint-points of TM helical proteins and tried to analyze the macroscopic arrangement or extension of TM helices by simplifying them as straight lines between joints. Thus, it should be noted that the measured dihedral angles cannot reflect the exact microscopic structural features of transmembrane segments because the transmembrane helices include kinks and bends^25, 26^. However, it is known that the bending angles of most TM helices are known to be comparatively low (less than 20 degrees) due to the limited membrane space^27, 28^, which indicates that the joint-based dihedral angle data would provide us a macroscopic viewpoint of angles between TM helices.

### Distribution of Ω and λ angles and their relevance to arrangement of helices

**Figure 3:**
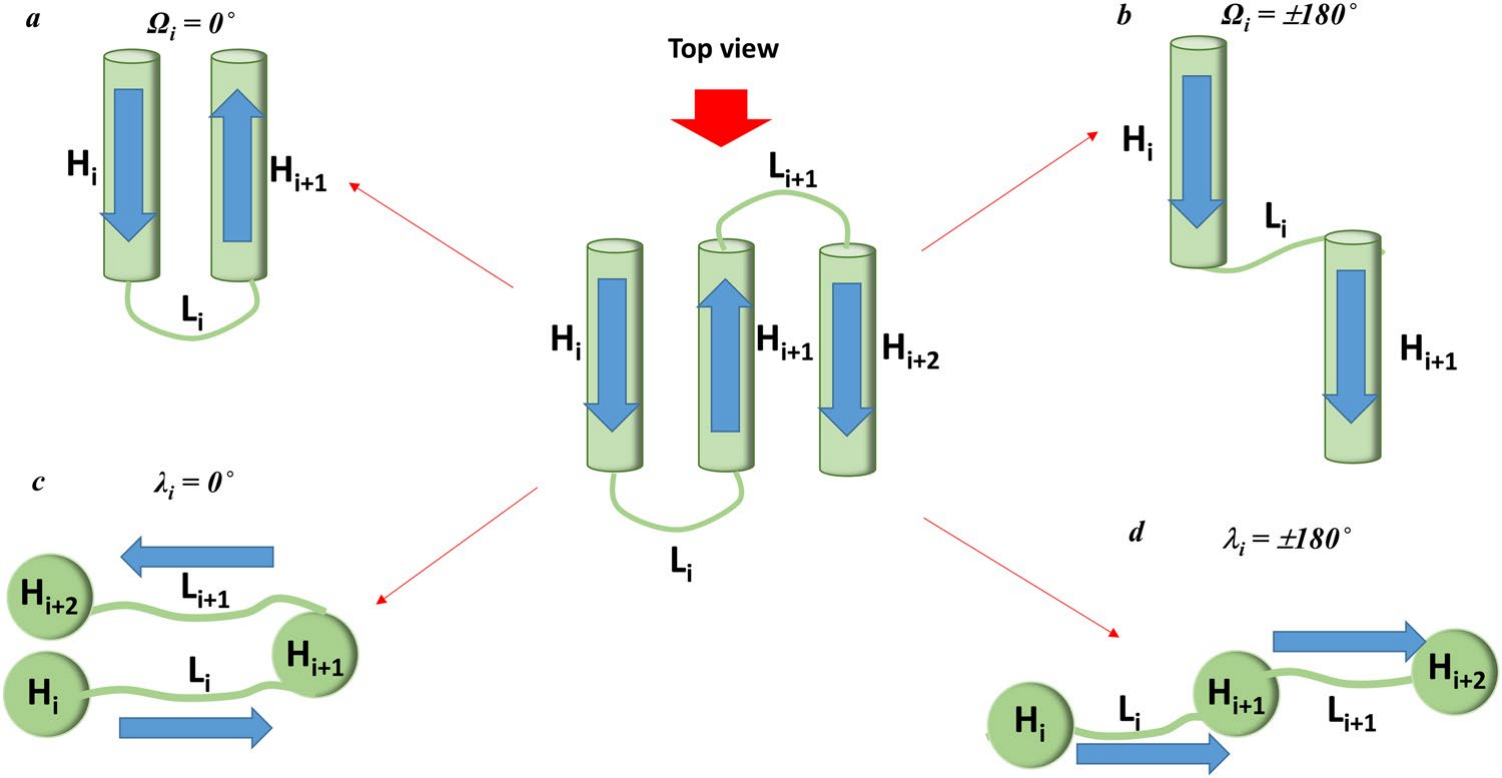
Arrangements of the helices and loops depending on the Ω and λ dihedral angles. The central figure presents the front view of three consecutive helices in the membrane proteins. H_(n)_ represents the helices and L_(n)_ represents loops. (**a**) Front view of the arrangement of two adjacent helices, H_i_ and H_i+1_ when Ω_i_ = 0°, (**b**) Front view of the arrangement of two adjacent helices, H_i_ and H_i+1_ when Ω_i_ =  ± 180°, (**c**) Top view of the arrangement of two adjacent loops, L_i_ and L_i+1_, and three adjacent helices, H_i_, H_i+1_ and H_i+1_, when λ_i_ = 0°, and (**d**) Top view of the arrangement of two adjacent loops, L_i_ and L_i+1_, and three adjacent helices, H_i_, H_i+1_ and H_i+1_, when λ_i_ = ±180°. These figures show the possible scenario depending on the dihedral angles rather than a real arrangement observed in TM proteins.

The dihedral angles between the joints are strongly related to the arrangements of the TM helices in membrane proteins at the macroscopic level. If the TM helices are simplified as straight lines of the joint points, as shown in the Fig. 2a, Ω type dihedral angles represent the arrangement of the TM helix region between the i^th^ TM helix (H_i_) and its adjacent i + 1^th^ TM helix (H_i+1_). The type λ dihedral angles also provides additional information of the relative arrangement between the i^th^ TM helix (H_i_) and i + 2^th^ TM helix (H_i+2_), considering that the i + 1^th^ loop (L_i+1_) is attached to the i + 2^th^ TM helix (H_i+2_). Figure 3 shows specific examples of the relationship between the dihedral angles and helical arrangements. When the dihedral angle Ω_i_ is close to 0°, helix H_i_ and the adjacent helix H_i+1_ are in an anti-parallel arrangement (Fig. 3a). On the other hand, when the dihedral angle Ω_i_ is close to ±180°, helix H_i_ and the adjacent helix H_i+1_ are in parallel (Fig. 3b). When the dihedral angle λ_i_ is close to 0°, helix H_i+2_ and helix H_i_ are in the same side with respect to helix H_i+1_ (Fig. 3c). On the other hand, when the dihedral angle λ_i_ is close to ±180°, helix H_i+2_ and helix H_i_ are in the opposite side with respect to helix H_i+1_ (Fig. 3d).

**Figure 4:**
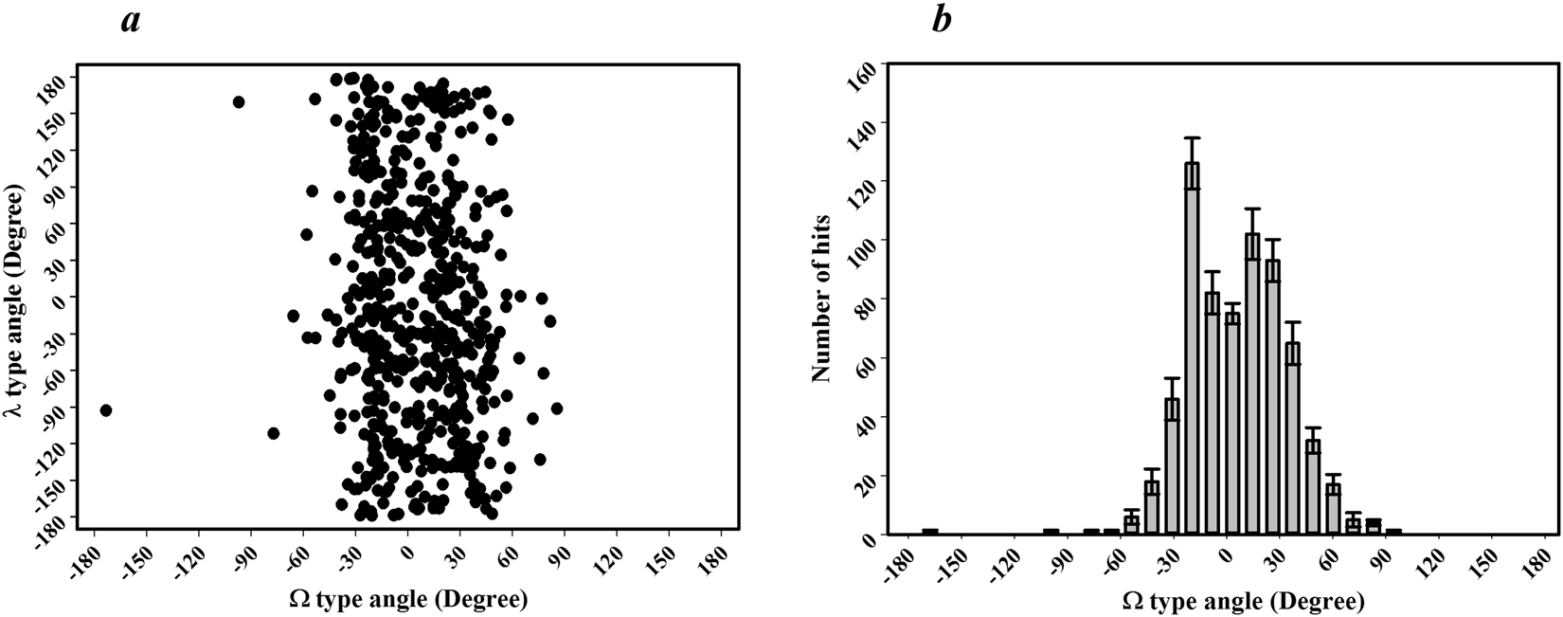
Distribution of the Ω type and λ type dihedral angles in the helical membrane proteins. (**a**) Ω-λ distribution plot for the helical membrane proteins. All Ω and λ type dihedral angles in the 103 non-homologous of membrane proteins are plotted together in the 2-D scatter plot (Ω type: x-axis, λ type: y-axis). (**b**) Histogram showing the overall distribution of the Ω type dihedral angles. Error bars were the standard deviations estimated by bootstrap method^44^, resampling the data 500 times with replacement and repeating the analysis.

The distribution of dihedral angles for the type Ω and λ of the 103 non-homologous protein structures were analyzed using the (Ω, λ) plot (Fig. 4a). Such analysis was expected to play the role of the Ramachandran-plot, which may be used to determine the allowed and disallowed conformations of the TM helices for the 103 non-homologous membrane proteins. The Ω type dihedral angles were restricted to a very narrow region in the range of *−*50° to + 50°. On the other hand, the λ type dihedral angles were distributed in the entire region between *−*180° to + 180°. For quantitative analysis, the histograms for the respective dihedral angles were plotted (Fig. 4b and Supplementary Information Figure S2). In the case of the Ω type dihedral angles, more than 90% of the angles were in the range, *−*40° to + 40° (Fig. 4b). In particular, the two dominant dihedral angle distribution region were observed around *−*30° to *−*10° and +10° to +30°, showing a symmetrical bimodal distribution. For the λ type dihedral angles, however, no clear dominant angle distribution region like Ω type angles was observed (Supplementary Information Figure S2).

According to Fig. 4, the Ω type dihedral angles showed the preference in the narrow range of *−*40° to + 40° as a dominant accessible region, suggesting that two neighboring TM helices (H_i_ and H_i+1_) tend to arrange in an anti-parallel manner, as shown in the Fig. 3a. In particular, the two major preferred regions around *−*30° to *−*10° and +10° to +30° with a relatively low frequency around 0° suggest that the most preferable arrangement of two neighboring helices is a slightly slanted anti-parallel arrangement. In the analysis of Ω angles (Fig. 4), an exceptional value (*−*173°) was observed, and identified as Ω_7_ of 3QNQA. The Ω value indicates that two consecutive helices (H_7_ and H_8_) of the protein are almost arranged in parallel as shown in Fig. 3b. This is the case that joint-based dihedral angle cannot reflect the exact structural features of transmembrane segments, which was mentioned above. It was confirmed that the H_7_ and H_8_ of the protein were a kinked helix and a short helix with long loop as a hugely bending TM segment, respectively, which resulted in such Ω value although the two consecutive TM regions are not parallel. On the other hand, the λ-type dihedral angles were distributed entirely in the all possible ranges of *−*180° to +180°. This suggests that helix H_i+2_ can be arranged randomly between the same side (Fig. 3c) and opposite side (Fig. 3d) to helix H_i_.

The dihedral angle analysis for the Ω-type suggested that the two adjacent TM helices prefer an anti-parallel orientation. Structurally, the helices that cross the hydrophobic lipid bilayer membranes prefer an anti-parallel arrangement. Thermodynamically, the anti-parallel arrangement of the two consecutive TM helices inside the lipid bilayer has stability by decreasing the internal energy due to a packing interaction. These are well-known features of helices in TM proteins^29–31^, which suggests that the joint-based approach is effective to explain the conformational features of TM proteins.

### Local pattern of consecutive Ω or λ angles and their relevance to extension of helices

As shown above, measurements of the dihedral angle Ω_i_ provides additional information about the arrangement of the neighboring TM helices (H_i_ and H_i+1_). The relative arrangement of the two TM helices H_i_ and H_i+2_ can be determined by measuring the dihedral angle λ_i_. This suggests that measurements of the consecutive dihedral angles can allow a prediction of how the TM helices in the membrane proteins are arranged sequentially or extended. For example, the information of Ω_i_ and Ω_i+1_ can determine the arrangement of H_i_, H_i+1_, and H_i+2_, and the information of λ_i_ and λ_i+1_ may allow a prediction of the relative positions of H_i+2_ and H_i+3_ to H_i_ and H_i+1_. The helical extensions in the membrane proteins were examined through a joint-based approach using the local patterns of the consecutive dihedral angle clusters, such as Ω_i_-Ω_i+1_, and λ_i_-λ_i+1_.

**Figure 5:**
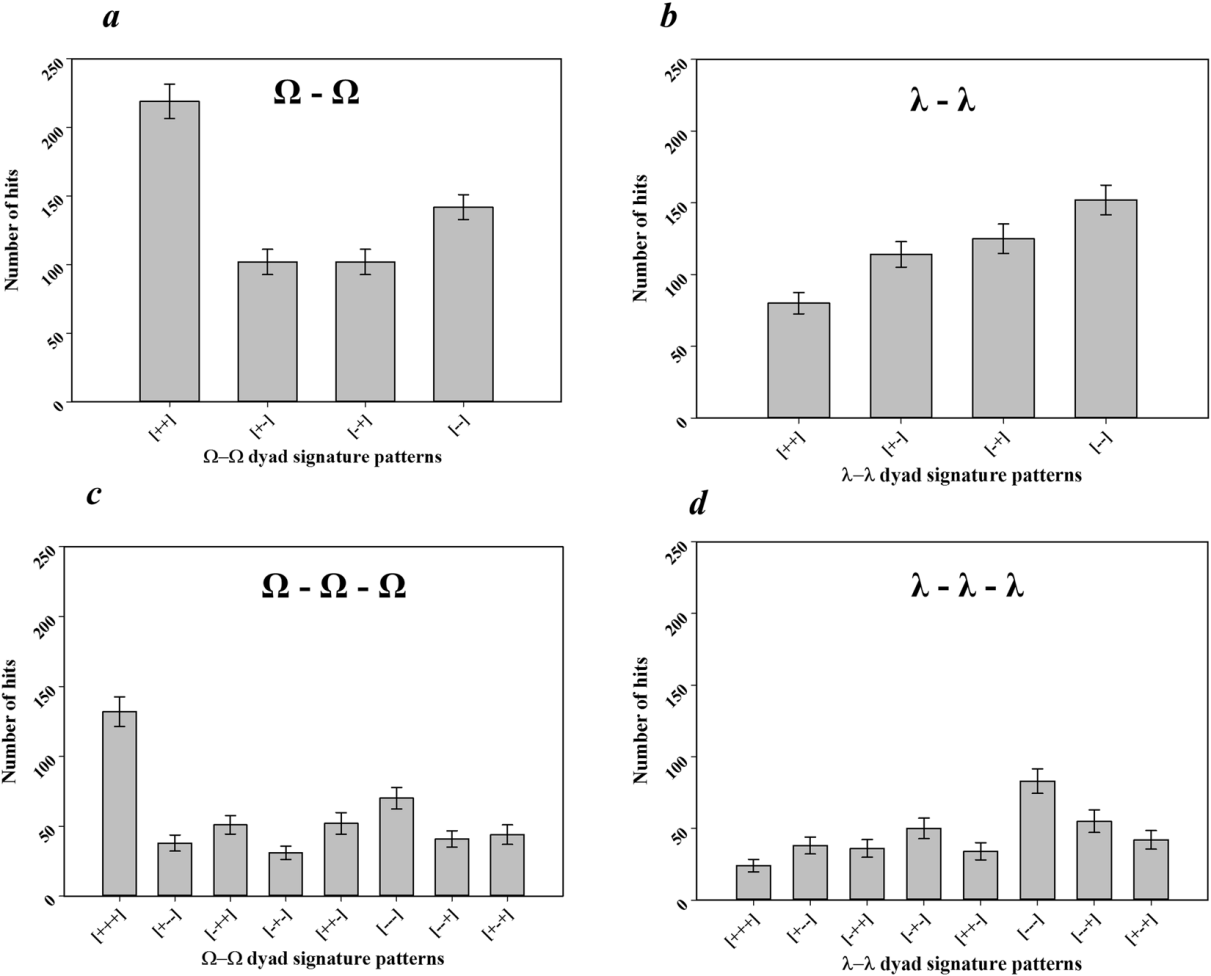
Frequencies of the patterns for consecutive Ω or λ type dihedral angles when Ω or λ type angles are categorized as (+) and (−). The bar diagrams show the observed numbers of (**a**) four different patterns of two consecutive Ω type angles, Ω_i_–Ω_i+1_ (**b**) four different patterns of two consecutive λ type angles, λ_i_-λ_i+1_ (**c**) eight different patterns of three consecutive Ω type angles, Ω_i_-Ω_i+1_-Ω_i+2_, and (**d**) eight different patterns of three consecutive λ type angles, λ_i_-λ_i+1_-λ_i+2_. For (**a**) to (**d**), (i) Ω or λ type dihedral angles were split into two regions, i.e., (+) = 0° to 180°, (−) = 0° to −180°, (ii) four and eight patterns were generated from the combinations of two consecutive angles, and three consecutive angles, respectively, and (iii) finally, the number of patterns in the 103 non-homologous proteins were measured. Error bars were the standard deviations estimated by bootstrap method, resampling the data 500 times with replacement and repeating the analysis^44^.

**Figure 6:**
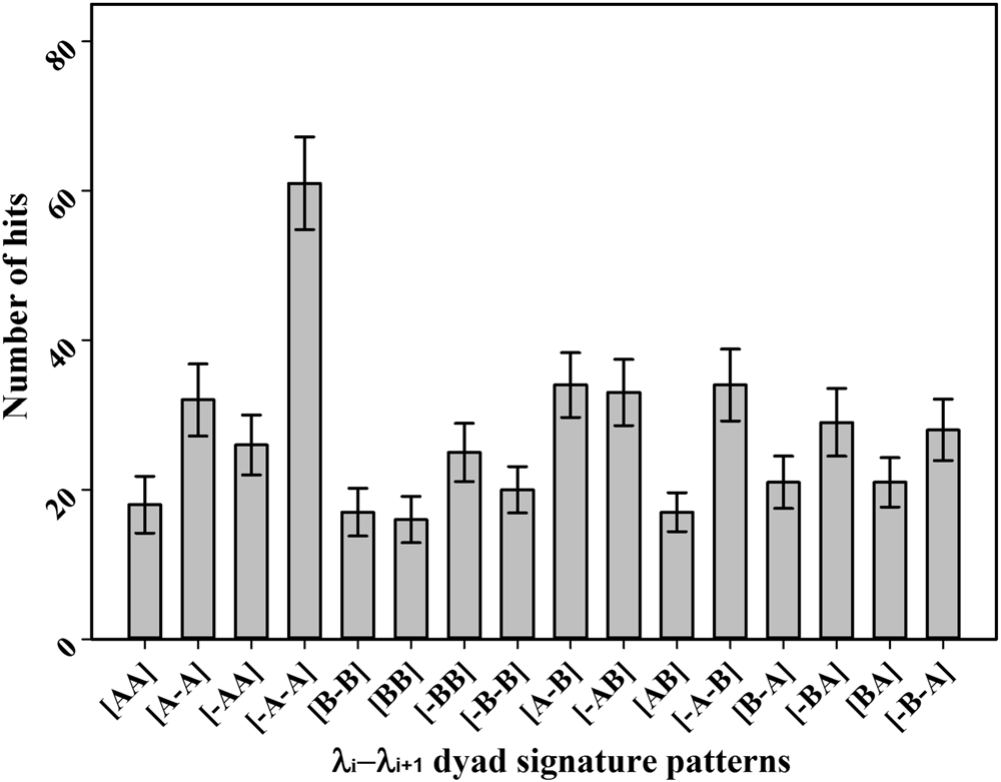
Frequencies of the patterns for consecutive λ type dihedral angles when λ type dihedral angle was split into four regions. The bar diagram shows the observed numbers of the 16 different patterns of two consecutive λ type angles, λ_i_–λ_i+1_. Here, all λ type angles were split into four regions, i.e. A = 0° to 90°, B = 90° to 180°, −A = −90° to 0°, and −B = −90° to −180°; 16 patterns were generated from the combinations for two consecutive λ type angles, λ_i_-λ_i+1_, and finally the numbers of the patterns in the 103 non-homologous proteins were measured. Error bars were the standard deviations estimated by bootstrap method, resampling the data 500 times with replacement and repeating the analysis^44^.

All dihedral angles were categorized into two groups: positive (clockwise) and negative (counter-clockwise) signs in a simple manner. The dihedral angles of Ω and λ for the 103 non-homologous proteins (Supplementary Information Table S1) can be represented as two signs, i.e., positive and negative (Supplementary Information Table S2). Combinations of the two signs can generate four patterns, i.e., (+, +), (*−*, *−*), (+, *−*), and (*−*, +), for the two consecutive dihedral angle clusters, such as Ω_i_-Ω_i+1_ and λ_i_-λ_i+1_. In a similar manner, eight patterns can be generated for the three consecutive dihedral angle clusters, such as Ω_i_-Ω_i+1_-Ω_i+2_ and λ_i_-λ_i+1_-λ_i+2_. All the frequencies of the patterns in the 103 non-homologous proteins were analyzed. For Ω_i_-Ω_i+1_ cluster, the frequency of the (+, +) pattern was the most dominant pattern (Fig. 5a), whereas frequency of the pattern (*−*, *−*) was the most dominant pattern for λ_i_-λ_i+1_ cluster (Fig. 5b). For the three consecutive dihedral angle clusters, i.e., Ω_i_-Ω_i+1_-Ω_i+2_ cluster and λ_i_-λ_i+1_-λ_i+2_ cluster, (+, +, +) and (*−*, *−*, *−*) were the most dominant patterns among the 8 possible patterns (Fig. 5c and d), respectively. As mentioned previously, the λ-type dihedral angles were distributed in the entire range from *−*180° to 180° (Fig. 4a), which motivated us to divide the dihedral angle space further into four quadrants, i.e., *−*180° to *−*90° (denoted as −B), *−*90° to 0° (denoted as −A), 0° to 90° (denoted as A), and 90° to 180° (denoted as B), and the pattern of the λ_i_-λ_i+1_ cluster was examined more in detail. Among the 16 possible patterns for the λ_i_-λ_i+1_ cluster, the most dominant distribution was observed in the range, *−*90° to 0° and *−*90° to 0° (Fig. 6).

**Figure 7:**
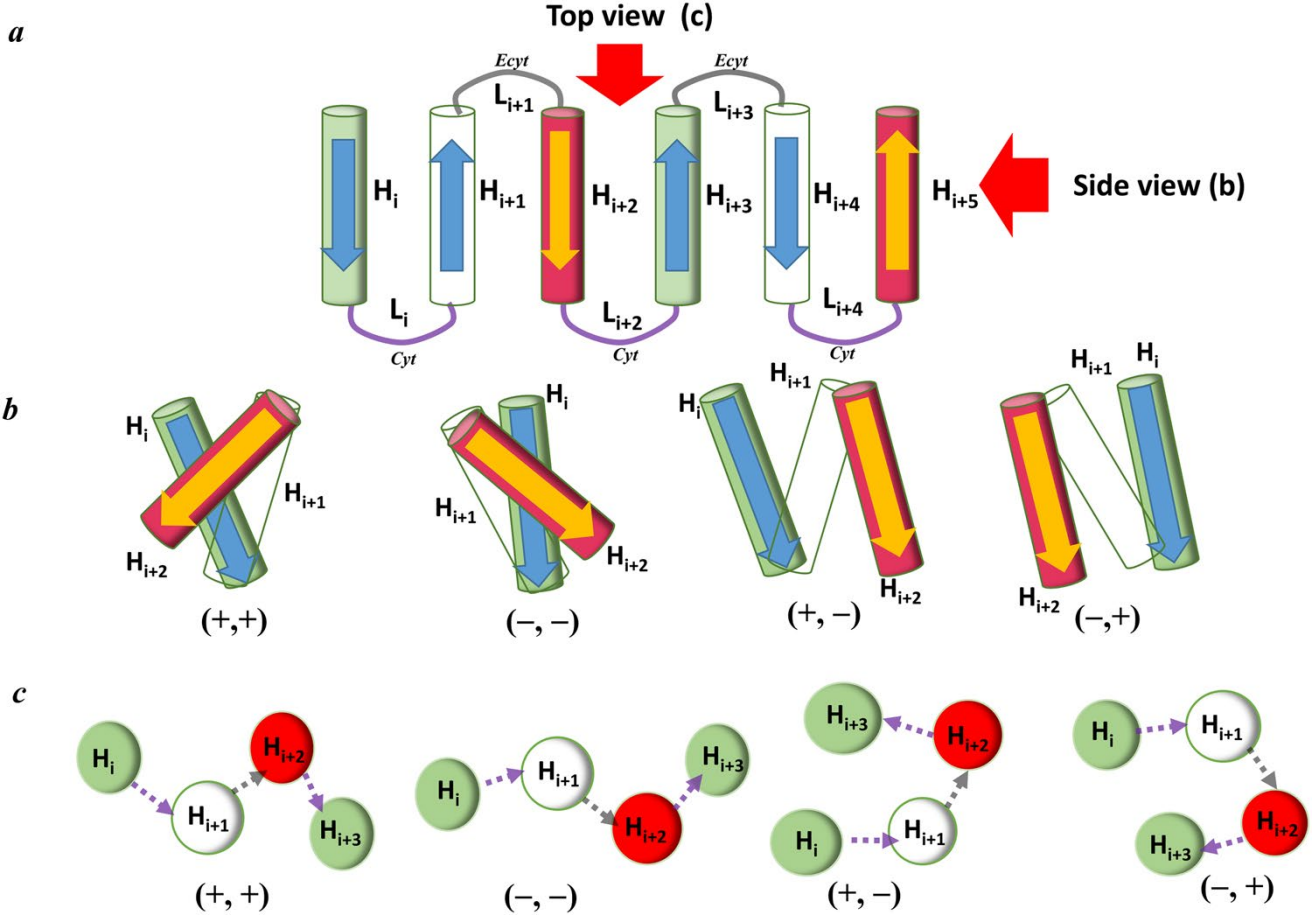
Relation of the dihedral angle patterns and helical arrangements or extensions. (**a**) Front view of the linearly ordered helical membrane proteins with the TM helices and loops. (**b**) Side view of the arrangement of three consecutive helices H_i_-H_i+1_-H_i+2_ depending on the four different patterns of two consecutive Ω type angles, Ω_i_-Ω_i+1_. (**c**) Top view configuration of four consecutive helices, H_i_-H_i+1_-H_i+2_-H_i+3_, for the four different patterns of two consecutive λ type angles, λ_i_-λ_i+1_. Figure 7(a) and (b) were generated as follows. (1) The dihedral angles were computed by the original definition in the Fig. 2, (2) arrangement of helices was predicted based on the dihedral angles, and then (3) the predicted arrangement was drawn based on side view and top position. Zig-zag conformation denotes the helices are arranged or extended in the alternative direction as depicted in ++ and −− configurations.

Figure 7a shows a schematic diagram of the membrane proteins with several TM helices observed in the front view. Figure 7b shows the schematic arrangement of three consecutive helices, i.e., H_i_, H_i+1_, and H_i+2_, depending on the pattern of the Ω_i_-Ω_i+1_ cluster, observed in the side view. As shown in the schematic diagram, four different (+, +), (+, *−*), (*−*, +) and (*−*, *−*) patterns determine four different types of configurations between H_i_ and H_i+2_ in parallel. The predominance of the (+, +) and (+, +, +) patterns in Ω_i_-Ω_i+1_ and Ω_i_-Ω_i+1_-Ω_i+2_ cluster analyses indicate that the membrane proteins favor the alternative packing of TM helices: zig-zag pattern. Figure 7c presents a schematic diagram of the membrane proteins with several TM helices observed in the top view and shows how the helices were extended depending on the pattern of the λ_i_-λ_i+1_ cluster. The (*−*, *−*) and (+, +) patterns suggest that the TM helices are extended in one direction with a zig-zag pattern. The (+, *−*) and (*−*, +) patterns, however, show that the TM helices are extended such that they are packed in a relatively compact space. According to the Fig. 5(b), (*−*, *−*) is the most preferred pattern, (*−*, +) and (+, *−*) patterns are next dominant patterns with similar frequency, and (+, +) is the least preferred pattern. The frequencies of the four patterns indicate that TM helices in the membrane proteins are extended by using zig-zag type extension and packing type extension almost equivalently. The large difference of frequency between (*−*, *−*) and (+, +) suggest that there is a significant directional bias in the zig-zag type extension, whereas there is no directional preference in the packing-type extension. The dominant pattern of (*−*90° to 0°, *−*90° to 0°) in the 16 possible patterns of the λ_i_-λ_i+1_ cluster (Fig. 6) suggests that there is also some angle preference between the TM helices in the extension of helices.

As shown in Fig. 7, both (+, +) and (*−*, *−*) patterns are equivalent in the point that they present the zig-zag pattern in the helical alignment (Fig. 7b) and helical extension (Fig. 7c). The only difference is that the (+, +) pattern is for a helical alignment and (*−*, *−*) is for a helical extension. The biased zig-zag patterns in the helical alignment and extension may be relevant to the stereochemistry of the residue-residue interaction between the TM helices and the interaction between the loops and the environment.

### Effect of TM helical position and number on the arrangement and extension of helices

As the number of TM helices increases, the arrangements of the TM helices in membrane proteins might be changed due to a change in the interaction energy term between the TM helices. To check this point, two kinds of analysis were carried out. First, the distributions of the dihedral angles for Ω and λ types were analyzed according to their relative position in the TM helices (Supplementary Information Figure S3(a) and (b)). The histograms for the distribution are shown (Supplementary Information Figure S1(a) and (b)). Ω_n_ or λ_n_ (n = 1, 2, 3, 4 …) denotes the n_th_ Ω or λ type dihedral angles in the 103 non-homologous proteins. The Ω type dihedral angles from Ω_1_ to Ω_11_ showed similar distributions and histograms in the range of approximately *−*50° to +50° (Supplementary Information Figure S3(a)). The λ type dihedral angles also exhibited a similar distribution and histogram patterns from λ_1_ to λ_10_, showing the distributions approximately in the entire ranges (Supplementary Information Figure S3(b)). These distribution patterns are similar to their overall distributions, as shown in Fig. 4. These results suggest that the relative arrangements of the two or three consecutive helices are not affected significantly by the relative position of the TM helices in the membrane. The distribution patterns of the terminal dihedral angles, such as Ω_12_, Ω_13_, λ_11_ and λ_12_ deviated substantially from the other ones, but an interpretation of such results may not be effective due to the insufficient sampling.

**Figure 8:**
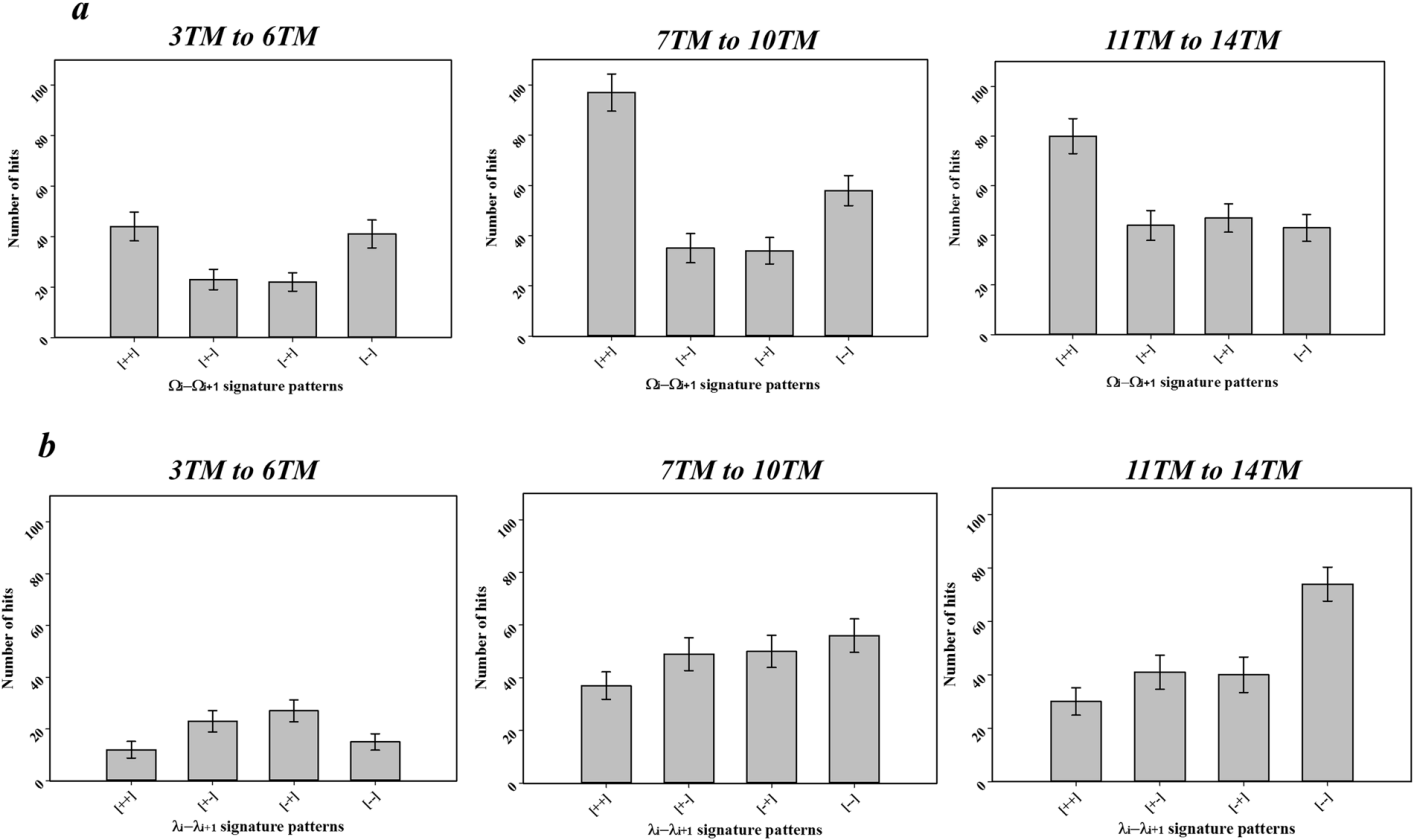
Frequencies of the patterns for two consecutive Ω or λ type dihedral angles depending on the proteins with a different number of TM helices. The 103 non-homologous proteins were categorized into three groups, i.e., proteins with 3 to 6 TM helices, 7 to 10 TM helices, and 11 to 14 TM helices. The bar diagrams show the observed numbers of (**a**) four different patterns of two consecutive Ω type angles, Ω_i_-Ω_i+1_, in each group, and (**b**) four different patterns of two consecutive λ type angles, λ_i_-λ_i+1_, in each group. Here, like (**a**) and (**b**) in the Fig. 5, (i) Ω or λ type angles were split into two regions, i.e. (+) = 0° to 180°, (−) = 0° to −180°, (ii) four patterns were generated from the combinations of two consecutive angles, and iii) finally, the number of the patterns in the three groups were measured. Error bars are generated by bootstrapping represent standard deviation, resampling the data 500 times with replacement and repeating the analysis^44^.

In addition, the frequencies of the four patterns for the Ω_i_-Ω_i+1_ or λ_i_-λ_i+1_ clusters were analyzed according to three different groups: proteins with 3–6 TM helices, proteins with 7–10 TM helices, and proteins with 11–14 TM helices. As shown in Fig. 8a, for the Ω_i_-Ω_i+1_ cluster, the (+, +) pattern shows up more frequently in the proteins with 3–6 TM helices. The pattern is roughly maintained as the TM number increases from 3–6 TM helices to 7–10 TM and 11–14 TM helices. These results indicate that the membrane proteins favor the alternative packing of TM helices in the helical arrangement regardless of their sizes. On the other hand, for λ_i_-λ_i+1_ cluster, (*−*, *−*) and (+, +) patterns show up less frequently than (+, *−*) and (*−*, +) patterns in the proteins with the 3–6 TM helices (Fig. 8b). As the number of TM helices increase, however, the (*−*, *−*) pattern becomes more dominant than other patterns. These results suggest that the TM helices prefer to be packed in their extension for small TM proteins, but zig-zag type extension plays an important role in the helical extension as the number of TM helices of the membrane proteins increases. Presumably, the zig-zag type extension of large TM proteins has the advantage of the efficient extension of the helices of large TM proteins in relatively narrow space inside the lipid bilayer.

### Identification of symmetric pairs in TM proteins

The analyses of consecutive Ω and λ angles shown in the Fig. 5 also indicate that there are many local symmetric configurations in the arrangement helices of membrane proteins. For example, (+, +, +) presents the symmetric configuration of (−, −, −). This observation motivated us to explore the existence of a symmetric configuration in the level of global TM protein structure. For this, we first assigned the proteins showing symmetric configurations based on λ angle signs from the whole TM protein dataset, and then selected protein pairs showing roughly symmetric configuration at a level of macroscopic 3-dimensional structure by visual inspection. λ angle sign was focused in the first step because λ angles showed more significant variations compared to Ω angles (Fig. 4) and therefore they may affect the 3-dimensional protein structure more. The proteins showing symmetric configurations based on λ angle signs were identified only in 3–6 TM proteins, and presented in Supplementary Information Table S3. In the dataset of 7 to 14 TM proteins, any protein pairs showing symmetrical property of λ angle signs were not detected, and further investigation for structural symmetry was not executed. Supplementary Information Table S4 shows the protein pairs exhibiting symmetry based on macroscopic 3-dimensional structure. Briefly, among the nine 3TM proteins in the whole dataset, two proteins (3ZE5A and 5AJIA) were identified to exhibit macroscopic 3-dimensional symmetry against three proteins (4O9PA, 3RKOA, and 1YQ3C). In the thirteen 4TM proteins, two proteins (4WD8A and 5DRIA) showed a symmetrical structure against one protein (1Q90A). In the nine 5TM proteins, 4A2NB and 3WVFA were identified as symmetric structural pairs. In 6TM proteins, some protein pairs showing symmetric configuration of λ angle signs were detected, but they were not structurally symmetric. Supplementary Information Figure S4(a) and (b) illustrates λ angle patterns and macroscopic helical arrangements of the three representatives symmetric protein pairs. These results indicate that there are protein pairs showing symmetric structural property in TM proteins, although the formation of symmetric pairs was not a general feature of TM proteins and observed only in small TM proteins. Further studies should be performed to understand the formation of such symmetrical pairs, but this study demonstrates that the joint-based approach can be efficiently used to find out some macroscopic structural patterns of TM proteins.

## Discussion

Examining the structural and conformational features of proteins in nature efficiently is still a challenging task because of their structural complexity and diversity. A macroscopic description of the protein structure offers a more simplistic way to understand structurally heterogeneous proteins, which can be complementary to the microscopic description method. In this report, a new macroscopic description method, i.e. joint-based description method, was introduced. The primary feature of the approach is to use a joint of secondary structures as the basic element for a description of the protein structure, whereas most developed protein structure description methods utilize physical entities, such as atoms, amino acids, and secondary structures. We performed the analyses of TM structures using the new joint-based approach, and found out some interesting conformational features or patterns in TM proteins. For example, we identified the allowed and disallowed regions of helical arrangement, variation of helical extension pattern depending on TM protein sizes and the possibility of structurally symmetric pairs in TM proteins. This study revealed a possible way to examine the arrangements of physical entities by investigating those of the joints between physical entities at the macroscopic levels. This study focused on membrane proteins, but the joint-based description method is expected to be applied to examine the conformational features of other classes of proteins and find the new features of protein structures in nature.

We note that there is no large difference in the angle distributions according to the configuration of TM helices for both the Ω-type and λ-type (Supplementary Information Figure S3a and b). The TM helices positioning in the membrane proteins can be restricted significantly inside the lipid bilayer as the number of TM helices increases, which might result in the variability of the dihedral angle distributions of Ω and λ-type according to their configurations. Surprisingly, these results suggest that the local arrangement of two consecutive TM helices is not affected too much by the position of the TM helices. On the other hand, an analyses of the patterns for Ω_i_-Ω_i+1_ and λ_i_-λ_i+1_ clusters revealed a clear preference of the zig-zag pattern in the packing and extension of the TM helices for the membrane proteins with high TM numbers (Fig. 8a and b). Presumably, this suggests that a zig-zag pattern is an optimized form required for the efficient TM helix-packing geometry inside the lipid bilayer. Overall, these results indicate that the membrane protein structure formations in the lipid membrane environment are controlled more significantly by an extension of the TM helix structures rather than the local arrangements.

A symmetric pair in the molecular geometry has been popularly observed in natural small molecules. Representative examples are the existence of stereoisomerism of amino acids and monosaccharides. Our joint-based approach allowed us to catch that there are some geometrically symmetric pairs in TM proteins. This suggests that the symmetric properties such as stereoisomerism observed in small molecules can exist in the level of global protein structures. Of course, this study was very limited to TM proteins and therefore further analyses should be performed against more expanded protein dataset. Our joint-based approach for protein structure is expected to be efficiently used in such studies.

Protein conformational diversity is closely associated with its functions. From the macroscopic analyses of TM topology in terms of Ω and λ angles, we could observe some structural features which can be related to functions. For example, the unique dihedral space (more ++ dyad signatures for Ω and more −− dyad signatures for λ angle) can be related to channeling activity. It has been reported that 11–14 TMH proteins, where “zig-zag” conformation is the most common, are mostly transporters and this conformation is required to form a channel for ion transport^32^. In a similar way, 7TM GPCR protein families showed dihedral angle deviations that can be also related to functional features. For the 7TM GPCR, *3* ^*rd*^ to *5* ^*th*^ omega angles showed significant differences than other omega angles where the most functionally important structural changes occurs according to the previous studies^33, 34^. These indicate that the joint-based macroscopic approach for protein structures can be used in the study on the structure/function linkage.

The joint-based approach is expected to be used for predicting the conformations of the transmembrane helices, a problem that can arise in low-resolution electron microscopy. In addition, it can be used for validating low resolution models of TM proteins similar to the previous studies such as “CaBLAM” method^35, 36^. As a further study, we have a plan to perform the applications of our approach to structural prediction and validation studies such as k-fold and leave-one-out cross validation based on machine-learning algorithm. For these applications, the joint-based dihedral angle determination method should also be further standardized since it can be sensitive to some factors such as definition of helices and accuracy of protein models.

Another potential of the joint-based metric is that it can be applied to new coordinates for molecular dynamics (MD) simulation of TM proteins at large scale. Membrane proteins are dynamic entities with partial folding and unfolding^23^. The computational time for folding and unfolding of complex membrane proteins at atomistic level is thus immense. Coarse-grained models such as MARTINI model^37, 38^ have been applied to MD simulation for the folding of membrane proteins within lipid bilayer, but they still have many limitations in computational time. In our joint-representation, a TM helix is treated as one unit of “rigid-body” at more coarse-grained model. Thus, a force field based on joint-representation can reduce the computational time scale to simulate the folding/unfolding of membrane proteins with large number of TM helices in lipid bilayer using molecular dynamics simulation. One of our long-term purposes is to develop an effective metric for such large scale coarse-grained MD simulation based on the joint-based approach.

## Methods

### Collection of structural dataset

First, with the aid of PDB, a search was made for membrane proteins with X-ray crystal structures and approximately 2600 structures were found. Only α helix containing proteins were then collected and separated to approximately 959 hits. The dataset of 511 refined structures having sequence identity less than 90%, including with resolution (≤3.5 Å) was selected for the unique proteins containing both homologous and non-homologous protein chains. Nearly 160 proteins with the sequence identity less than 30% with ≤3.5 Å resolution structures were extracted using PICESES server^39, 40^ and grouped as non-homologous membrane proteins. Helical proteins were classified according to the TM numbers from 3TM to 14TM. 55 protein structures in the same superfamily were treated as remote homologous and were expelled from the list. When choosing a monomer, only one conformation was considered where more than one conformation is available for the same superfamily. 103 protein chains were finally identified as a training dataset. To validate the completeness of the selected dataset, we performed DALI search using the selected 103 non-homologous proteins and examined how many structural homologs of the 103 proteins were detected in the whole 959 proteins. The 103 structures detected 89.7%, 96.2%, and 97.5% of the 959 proteins when the threshold of RMSD for structural homology was set to 3.0 Å, 4.0 Å, and 5.0 Å, respectively.

### Determination of structural joint points

To select the structural joints, the amino acid position was scrutinized visually for Cα XYZ coordinates from the corresponding PDB file. The written PYTHON program read each protein structure for the “HELIX” in the PDB files to detect their each helix residue and output their amino acid positions and Cα XYZ coordinates. In addition, specialized databases for the TM helices were also cross checked for their beginning and ending residue position numbers. For each individual protein, the specialized databases, such as OPM^41^, PDBTM^42^, and TMPad^43^, were referred to classify their SSE (Secondary Structure Element) topologies and were used to identify their helical and loop segments based on the coordinates obtained from PDB. To select the fixed joint points, we majorly relied on OPM helical segments annotation with the help of manual inspections to avoid ambiguities. Such specified residue coordinates for each secondary structure, i.e., helices, were treated as the structural joining points to represent protein macroscopically. Table 1 lists the PDB codes and the corresponding topology of the membrane proteins. The listed coordinates of the structural joints represent each SSE; their continuous adjacent joint points were chosen for each helix and loop. While establishing a connection of these joints residues, a new description of the overall protein structure was portrayed.

### Dihedral angle calculation of Ω and λ types

The filtered PDB structures were parsed and Cα XYZ coordinates preselected from each joint were exploited for the dihedral measurements. The first dihedral angle involving four joints P_1_, P_2_, P_3_ and P_4_ can be ascertained by measuring the angle between the two planes made by P_1_, P_2_, P_3_ and P_2_, P_3_, P_4_. Similarly, the second dihedral angle can be found by applying the structural points (P_2_, P_3_, P_4_, and P_5_), and the (P_3_, P_4_, P_5_ and P_6_) joints are used to determine the third. Initially, for the set of four *xyz* coordinate points that define a dihedral angle, the algorithm calculates three vectors, namely, $\overrightarrow{{V}_{1}}={P}_{1}-{P}_{2}$, $\overrightarrow{{V}_{2}}={P}_{2}-{P}_{3}$ and $\overrightarrow{{V}_{3}}={P}_{3}-{P}_{4}$, where $\overrightarrow{{V}_{n}}={P}_{x}-{P}_{y}$ is the vector from point **x** to point **y**. $\overrightarrow{{{\rm{V}}}_{1}}$ and $\overrightarrow{{{\rm{V}}}_{2}}$ defines the 1^st^ plane (Orthogonal frame, M_n_), whereas $\overrightarrow{{{\rm{V}}}_{2}}$ and $\overrightarrow{{{\rm{V}}}_{3}}$ does the 2^nd^ plane. The angle between these planes reflects the dihedral angle between the helices (or loops), which is designated as Ω (or λ). The normal unit vector to this plane was calculated by taking the cross product of these two vectors: $\overrightarrow{{{\rm{N}}}_{321}}=\frac{\overrightarrow{{{\rm{V}}}_{1}}={{\rm{P}}}_{1}-{{\rm{P}}}_{2}\ast \overrightarrow{{{\rm{V}}}_{2}}={{\rm{P}}}_{2}-{{\rm{P}}}_{3}}{|\overrightarrow{{{\rm{V}}}_{1}}={{\rm{P}}}_{1}-{{\rm{P}}}_{2}\ast \overrightarrow{{{\rm{V}}}_{2}}={{\rm{P}}}_{2}-{{\rm{P}}}_{3}|}$. The normal unit vector to the plane defined by the second, third and fourth joint coordinates $\overrightarrow{{N}_{321}}$ was calculated in an analogous manner. The angle between such planes reflects the dihedral angle between the helices, which is designated as *Ω*. The *arctan2* of *Ω* is calculated using the following relation: *dihedral*_1 = *np*.*arctan*(*Y*_1_, *X*_1_) These are combined and the angle is calculated using the *arctan2* function. Such measurements are converted from radians to degrees within the range of −180° to 0° to 180° using the following equation, *In*_*degrees*_1 = *dihedral*_1 ∗ 180°/*π* to facilitate the analysis. The resulting number of dihedral angles for each protein is directly proportional to the number of helices and loops present in them. A python script was developed in house and executed for the dihedral angle calculation using the Spyder python interface.

### Analyses of consecutive dihedral angle patterns

To perform the conformational search based on the signature patterns, the preferred orientations among various combinations of consecutive dihedral angles were counted statistically. For the 103 structures selected, each structure was presented by the Ω_n_-λ_n_-Ω_n+1_-λ_n+1_ dihedral angle sets, as summarized in Supplementary Information Table S1; n stands for Helix numbers in the protein structure. The calculated dihedral angles were converted to positive (+ve) and negative (−ve) signatures to represent the conformations, as given in Supplementary Information Table S2. A consecutive Ω-Ω pattern was selected for each fold as Ω_n_-Ω_n+1_. Grouped Ω_n_-Ω_n+1_ should be a consecutive, adjacent set, and no fixed order, whereas non-consecutive Ω_n_-Ω_n+2_ were not considered. For example, the Ω-Ω pattern angles were selected from Ω_1_-λ_1_-Ω_2_-λ_2_-Ω_3_-λ_3_ to Ω_n_-λ_n_ as any consecutive Ω_n_-Ω_n_. To make more defined distribution patterns, the consecutive Ω_n_-Ω_n+1_ and Ω_n_-Ω_n+1_-Ω_n+2_ were also tested.

## Electronic supplementary material

Supplementary Information
