# Supplementary material for: Joint-based description of protein structure: its application to the geometric characterization of membrane proteins
Source: Sci Rep. 2017 Apr 21;7:1056. doi: 10.1038/s41598-017-01011-z (PMC5430719; doi:10.1038/s41598-017-01011-z)
Supplement: Supplementary file 1 — Supplementary Information [file 41598_2017_1011_MOESM1_ESM.pdf]

Supplementary Information

Joint-based description of protein structure: its application to the geometric characterization of membrane proteins

Jayaraman Thangappan[a], Sangwook Wu[b,*] and, Sun-Gu Lee[a,*]

[a]Department of Chemical Engineering, Pusan National University, Busan, 609-735, Republic of Korea

[b]Department of Physics, Pukyong National University, Busan, 608-73, Republic of Korea

Figure S1: Histograms for the dihedral angles depending on their configurations. (a) Histograms for the Ω type dihedral angles depending on their configurations. The histogram for $\Omega_{(n)}$ shows the distribution of all $\Omega_{(n)}$ angles in the analyzed membrane proteins.

a

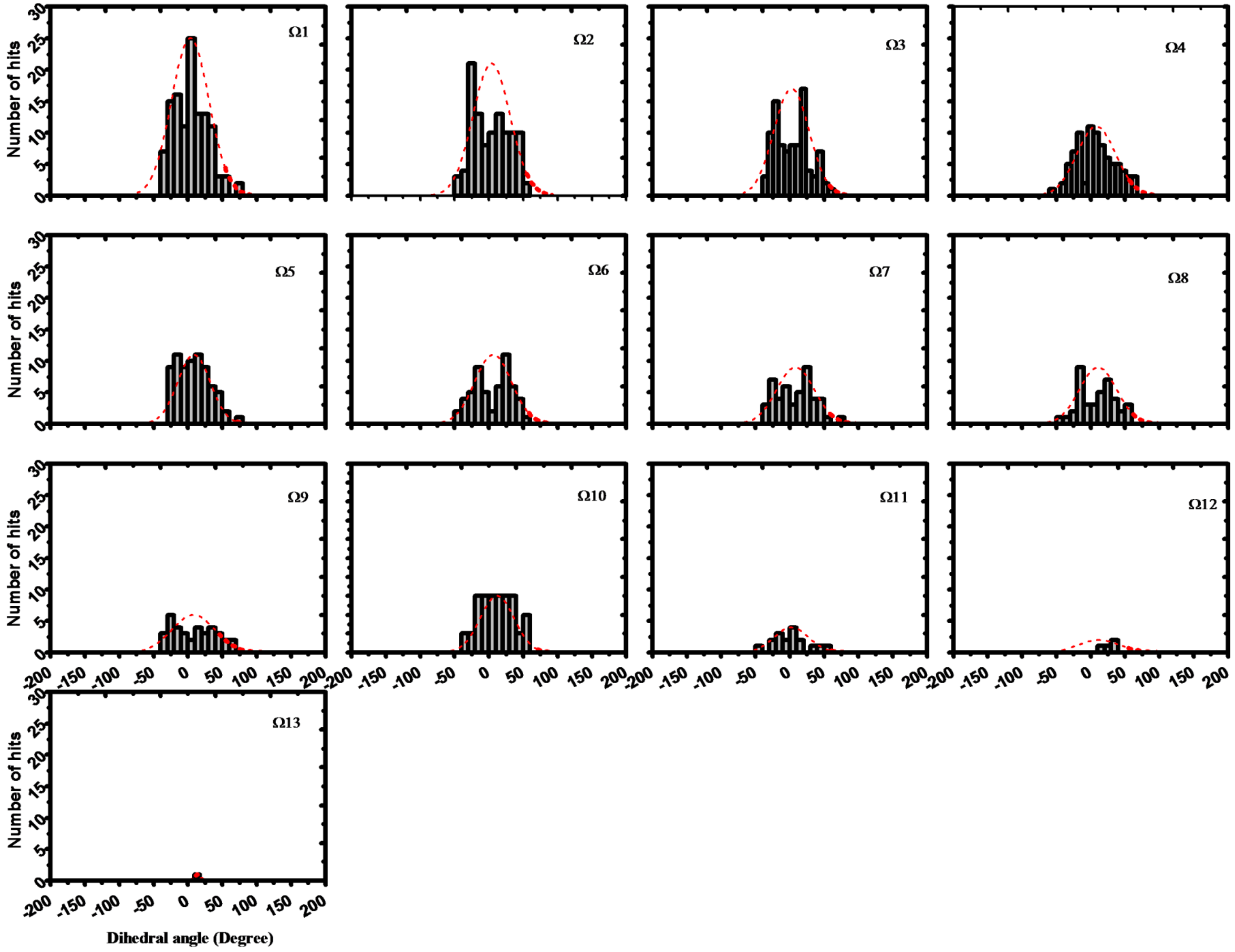
Ω1
Ω2
Ω3
Ω4
Ω5
Ω6
Ω7
Ω8
Ω9
Ω10
Ω11
Ω12
Ω13
Number of hits
Dihedral angle (Degree)

Figure S1: Histograms for the dihedral angles depending on their configurations. (b) Histograms for the λ type dihedral angles depending on their configurations. The histogram for $\lambda_{(n)}$ shows the distribution of all $\lambda_{(n)}$ angles in the analyzed membrane proteins.

b

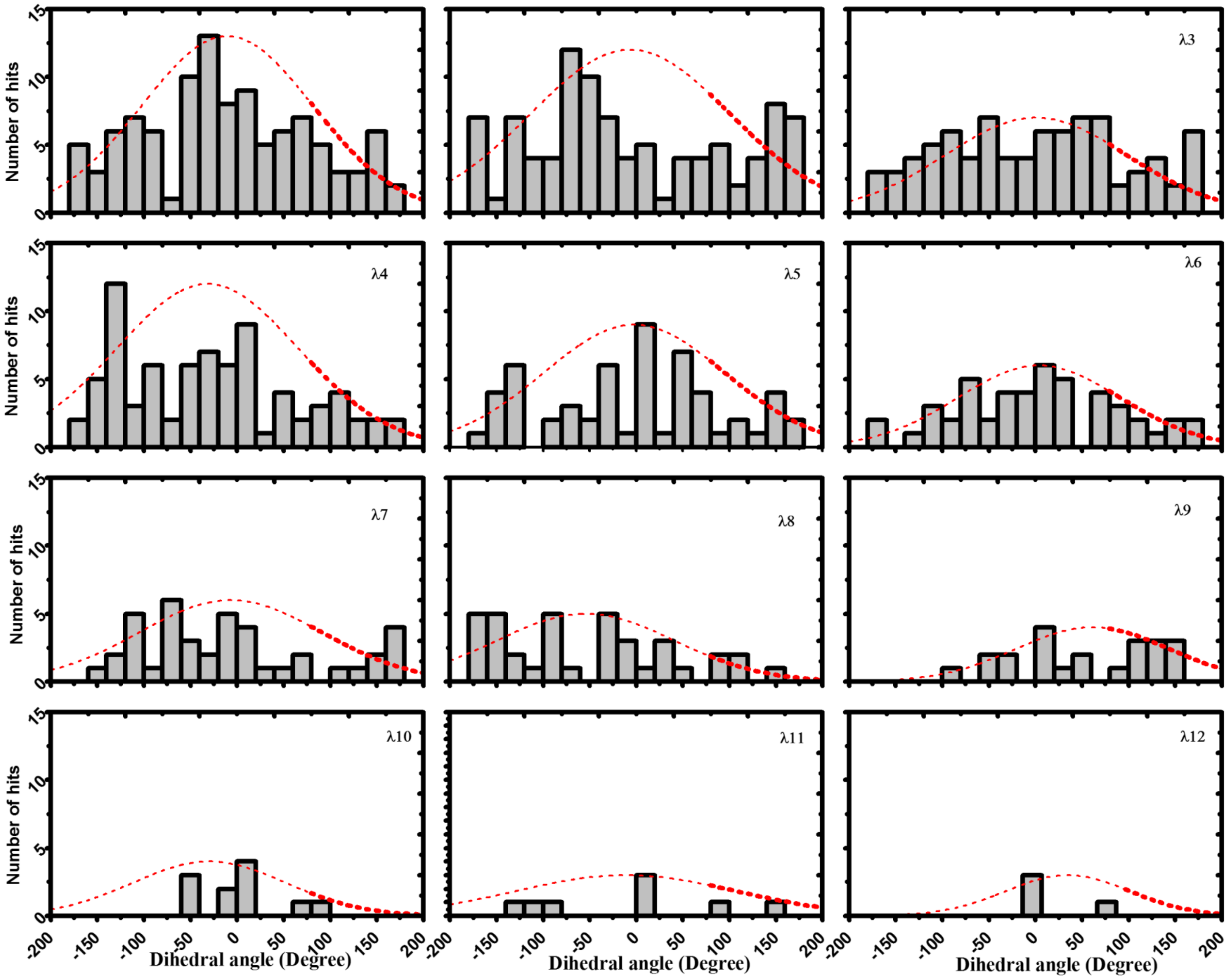

Number of hits
λ3
λ4
λ5
λ6
λ7
λ8
λ9
λ10
λ11
λ12
Dihedral angle (Degree)

Figure S2: The overall distribution of the λ type dihedral angles. Histograms for the λ type dihedral angles showing the distribution of all $\lambda_{(n)}$ angles in the analyzed membrane proteins.

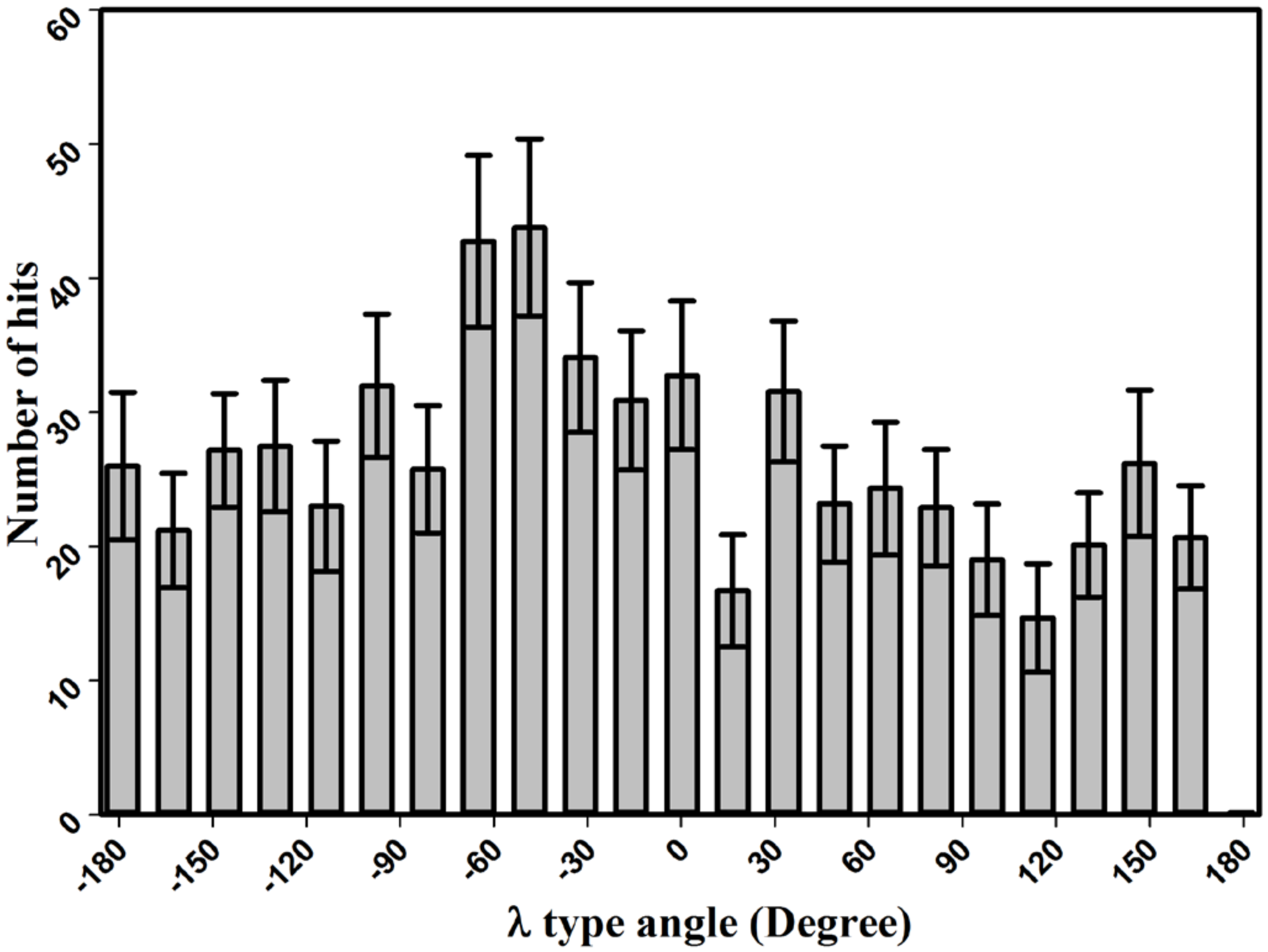

Figure S3: Dihedral angle distributions in the helical membrane proteins depending on their configurations. (a) Scatter plot of the Ω type dihedral angles depending on their configurations. (b) Scatter plot of λ type dihedral angles depending on their configurations. For (a) and (b), all the ith dihedral angles (Ω_i or λ_i values) in the 103 non-homologous proteins were collected from Supplementary Information Table 1, and plotted against Ω_i or λ_i in x-axis of (A) or (B).

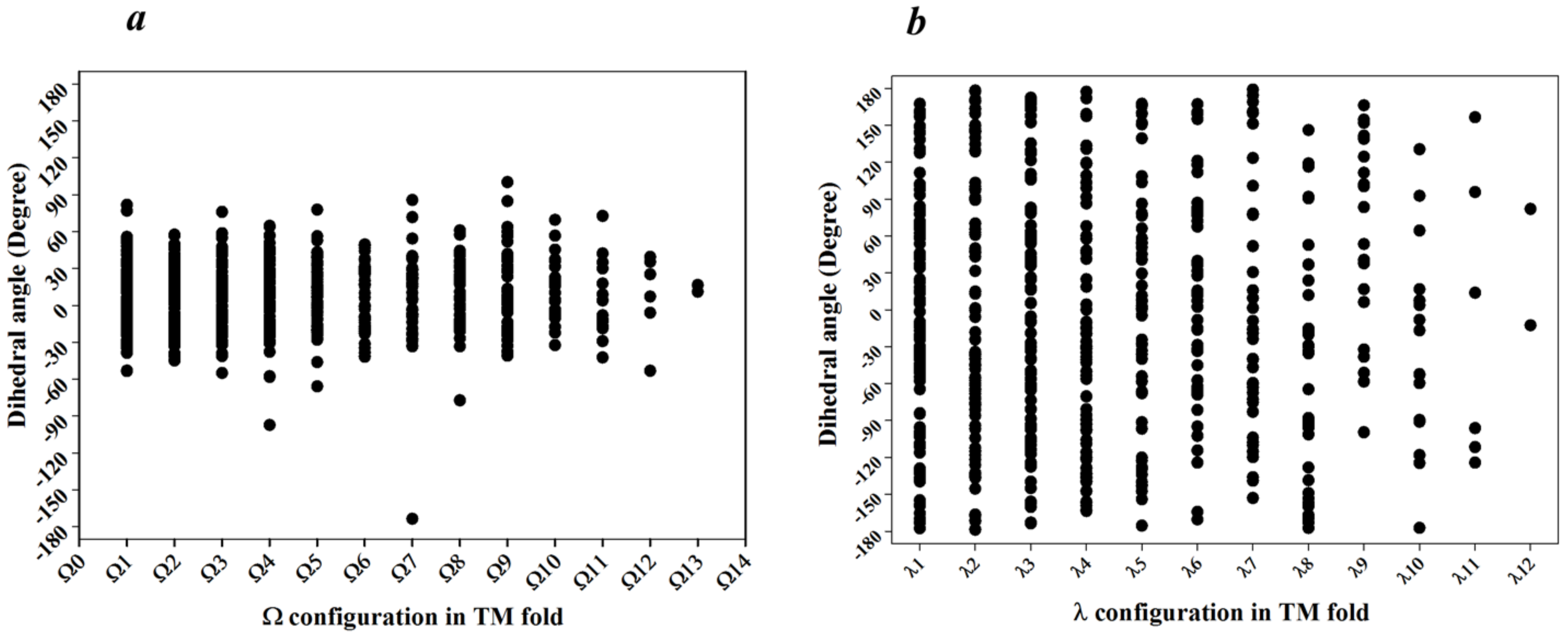

Figure S4: Identification of symmetric patterns in small TM proteins. (A) Ω-λ plots show symmetric pattern pairs of the small TMH protein pairs, i.e. 3TM-3ZE5:4O9P; 4TM-5DIR:1Q90 and 5TM-4A2N:3WVF (Ωn & λn: x-axis, Ω & λ angles in degree: y-axis). (B) 3D representations of symmetric patterns of the selected pairs are given with their respective PDB IDs.

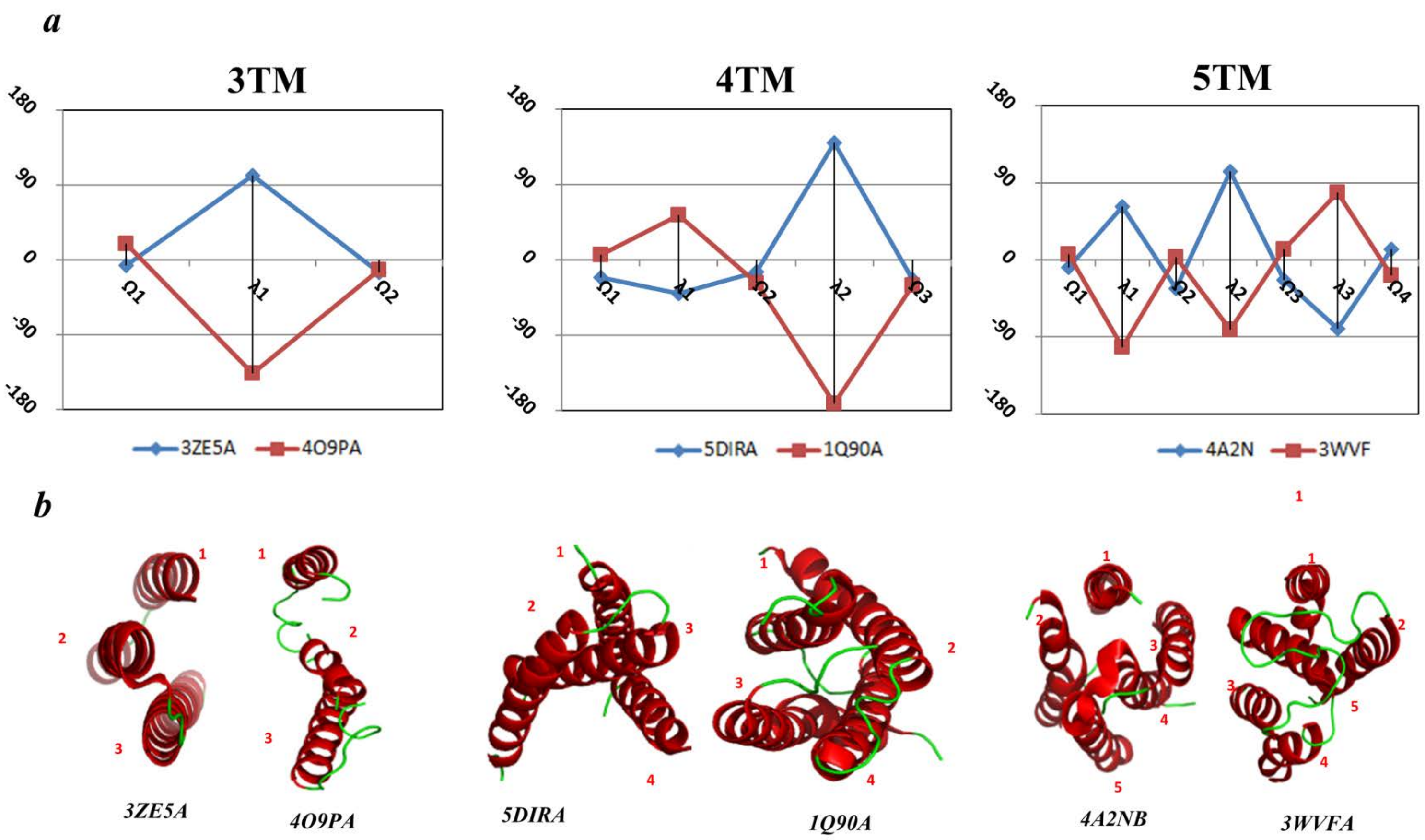

Table S1: Measured Ω and λ type dihedral angles for all 103 selected proteins.

#TM	PDB	Ω1	λ1	Ω2	λ2	Ω3	λ3	Ω4	λ4	Ω5	λ5	Ω6	λ6	Ω7	λ7	Ω8	λ8	Ω9	λ9	Ω10	λ10	Ω11	λ11	Ω11	λ12	Ω13
3TM	2ZT9B	21.65	69.71	-22.17																						
3TM	2BHWA	26.76	-17.02	-27.05																						
3TM	3ZE5A	-6.88	101.25	-15.03																						
3TM	4O9PA	19.13	-136.73	-12.27																						
3TM	1YQ3C	-38	-169.89	14.65																						
3TM	4X5MA	-21.08	8.47	-18.58																						
3TM	3RKOA	1.84	-159.38	-2.33																						
3TM	5AJIA	1.6	143.58	47.23																						
3TM	4U1WA	-16.74	-35.98	6.89																						
4TM	4HKRA	-19.51	111.3	-26.04	145.78	-5.55																				
4TM	2BL2A	-4.11	46.14	5.2	46.64	-0.8																				
4TM	4YMKA	-22.78	97.86	-25.19	-96.88	14.85																				
4TM	2UUHA	-3.92	65.72	18.09	60.83	-13.68																				
4TM	5TCXA	-14.03	-84.59	-41.34	-0.48	-17.39																				
4TM	5ER7A	-31.52	127.56	8.96	-54.72	-30.76																				
4TM	4WD8A	20.23	36.09	-25.01	-134.52	27.43																				
4TM	1KQFC	-10.61	18.27	-26.22	-135.84	-17.89																				
4TM	4RI2A	-15.02	-11.76	-22.91	171.05	-31.19																				
4TM	1Q90A	-20.82	-40.11	-14.13	139.78	-23.36																				
4TM	3EAMA	3.29	41.51	14.67	-126.14	-19.46																				
4TM	5DIRA	6.28	53.64	-27.43	-171.42	-30.75																				
4TM	2ZUQA	-5.48	-177.57	-15.13	-24.13	-25.15																				
5TM	4UC1A	20.15	67.77	-17.31	144.36	-20.38	-30.15	-19.3																		
5TM	3TUIA	22.28	-14.19	3.66	163.68	17.51	152.31	7.59																		
5TM	4A2NB	-8.6	62.3	-33.22	103.33	-23.68	-80.62	12.26																		
5TM	1Q16C	44.99	-173.21	21.46	14.91	18.76	129.62	22.82																		
5TM	3WU2A	-18.56	-84.03	-27.24	177.36	-12.8	63.28	-8.72																		
5TM	3WVFA	6.69	-101.99	2.75	-81.27	12.48	78.56	-18.42																		
5TM	3RGBC	-8.68	-11.51	-18.55	43.27	-25.1	-26.9	-21.13																		
5TM	4U9NA	53.37	34.18	20.36	-178.56	15.22	45.52	12.85																		
5TM	4NV5A	-17.39	-158.21	-20.75	-0.22	-29.98	-5.44	32.84																		
6TM	3RGBB	34.6	-129.07	35.74	-132.56	5.13	56.28	6.16	133.48	40.73																
6TM	4MRSA	-12.5	-23.44	24.91	-171.76	40.83	42.7	8.56	-139.26	15.89																
6TM	3UX4A	-20.67	-31.44	-21.16	178.18	-21.73	-49.42	-14.16	-133.02	-21.25																
6TM	4B4AA	18.39	7.07	45.64	-44.32	16.21	58.06	-3.12	-137.11	32.54																
6TM	3H90A	-28.43	149.49	9.48	-34.27	-28.8	39.67	-13.59	-139.84	-5.96																
6TM	5JWYA	13.23	-103.17	29.52	-5.79	44.4	-157.15	-30.22	4.42	33.86																
6TM	5I32A	22.12	-100.28	37.69	-121.64	36.05	82.86	-20.03	-14.37	26.48																
6TM	4P6VE	50.76	81.54	23.9	-166.52	32.05	36.77	35.87	-34.82	43.22																
6TM	3RVYA	-32.99	-9.85	-22.66	-178.81	-31	121.57	29.22	99.07	-8.11																
6TM	4O6MA	-5.1	-32.49	-6.7	-145.47	7.57	35.86	6.5	-25.84	38.74																
6TM	2XOWA	12.5	6.3	47.65	-35.81	55	126.92	-37.86	24.9	-24.38																
6TM	4XU4A	22.84	-54.84	49.77	65.58	10.21	-97.23	24.64	-38.5	14.28																

6TM	1OKCA	15.65	-33.16	16.48	50.02	-0.52	-40.39	14.89	57.68	6.8												
6TM	3RLBA	-11.7	-64.7	28.08	-133.14	13.95	54.37	12.58	86.42	12.02												
6TM	4O6YA	-14.16	-139.52	-8.26	-64.48	-22.42	164.22	-19.55	-121.61	8.75												
6TM	3WU2B	-15.67	102.14	9.43	-136.63	-23.01	106.92	-0.76	-156.99	-19.64												
6TM	3B4RB	12.69	58.22	39.52	-77.31	-19.73	170.11	63.93	-70.42	-17.31												
6TM	2R9RB	-34.6	-1.4	-41.06	169.49	-24.21	135.21	43.68	103.37	-2.33												
7TM	2Z73A	-13.74	-108.73	-14.17	146.87	20.82	16.27	1.73	-30.09	-20.11	-142.54	-0.017										
7TM	5SYTA	15.6	71.96	26.67	150.11	17.02	61.06	27	177.19	21.25	3.06	16.07										
7TM	4PGRA	28.77	-45.44	-12.03	-86.02	14.3	54.04	2.03	48.15	-17.99	-39.98	-23.17										
7TM	2DYRC	-31.64	24.85	-32.45	-72.54	-4.41	110.4	-17.78	-19.41	-28.08	159.26	-20.95										
7TM	5CTGA	-20.65	37.2	-22.25	31.58	-7.54	38.3	27.59	-129.01	-20.43	1.42	-18.72										
7TM	5AZBA	36.9	15.52	31.33	-178.59	19.09	-123.99	-25.25	91.61	7.19	-129.61	-19.57										
7TM	5EGIA	55.67	-111.41	27.92	97.13	-30.79	-113.72	56.39	-108.88	23.37	103.54	10.18										
8TM	5DWYA	-24.57	-38.54	-10.49	-71.14	45.98	5.32	19.77	-56.24	-6.15	45.26	26.75	-124.21	10.41								
8TM	2VPZC	-24.54	5.27	-27.94	177.99	-11.52	-139.69	42.67	-147.65	-16.42	0.42	-17.9	160.86	-28.9								
8TM	4QTNA	5.51	-154.93	-39.96	-45.3	-17.06	167.43	28.11	-105.84	29.55	-175.47	-41.22	-69.01	-23.06								
8TM	4J7CI	32.35	-111.3	-20.41	163.32	10.65	-160.35	10.42	118.7	-6.2	152.08	48.88	80.91	29.51								
8TM	3RFUA	-29.29	-35.54	-19.82	91.19	-5.95	24.22	6.63	-19.12	14.78	29.5	20.29	11.85	15.32								
8TM	4P02A	-30.58	66.87	-29.94	-59.64	-9.07	163.07	-23.91	0.04	42.58	-4.65	29.65	31.66	-27.51								
8TM	3TIJA	20.94	59.38	56.76	-68.2	21.47	62.86	11.21	-89.44	52.78	-58.31	26.4	86.98	37.74								
9TM	4O9PB	-17.57	-130.92	1.19	89.99	6.04	-117.16	-19.87	-115.67	13.1	54.68	-17.15	-66.09	5.8	159.83	-11.44						
9TM	4TQ4A	14.61	-172.81	-24.86	89.3	-39.32	-51.99	-12.19	159.14	24.29	167.39	-15.97	-28.73	-6.56	151.26	-7.54						
9TM	4Q2GA	-3.24	131.12	19.5	48.17	31.62	-124.85	9.73	119.49	56.32	6.89	29.01	167.15	30.35	-15.96	19.28						
10TM	2ZXEA	-28.14	82.68	-10.51	1.24	16.2	17.87	3.1	18.77	7.01	-28.31	36.84	-57.27	25.22	123.41	0.37	-64.64	37.81				
10TM	3QNQA	16.42	167.32	57.44	-36.27	23.78	-62.88	50.78	-31.52	2.49	-24.31	-38.52	-8.24	-173	168.93	27.74	52.73	12.08				
10TM	4P6VB	34.32	-98.92	40.91	-39.82	35.64	-9.93	56.9	171.86	-0.9	-66.48	-2.88	39.71	21.66	15.89	7.37	11.97	13.51				
10TM	4QUVA	76.94	-1.57	48.94	-104.22	44	-51.16	0.01	-133.65	-19.43	50.73	-0.35	37.94	-13.56	77.09	-26.71	-138.54	11.55				
10TM	5I20A	32.54	-137.97	13.4	62.95	18.87	172.4	2.2	-40.66	42.87	76.46	16.85	-102.52	17.09	100.81	19.21	-177.37	-24.14				
10TM	2NQ2A	37.09	138.25	-25.15	70.17	17.92	-96.27	33.61	-97.49	-0.96	19.74	-18.94	-81.45	47.07	-67.43	30.73	-148.92	51.86				
10TM	3V5UA	25.74	-95.5	28.28	-55.85	-10.9	68.71	7.69	-29.45	35.77	40.68	31.43	-114.32	4.41	-60.69	-13.44	-153.31	-5.58				
10TM	4WISA	14.03	-133.36	33.19	100.35	6.64	-88.4	42.31	-138.87	8.65	-33.26	-22.55	-33.63	-7.66	-8.93	10.89	-172.81	32.44				
10TM	3M73A	-23.96	13.77	-22.31	-64.46	-9.29	-9.26	-13.08	-70.49	-4.54	11.66	-8.82	-45.12	-9.17	-18.88	-18.12	-35.23	-17.16				
10TM	4N7WA	7.15	-95.5	17.83	-55.85	44.25	68.71	10.35	-29.45	16.94	40.68	21.43	-114.32	44.22	-60.69	55.35	-153.31	-13				
10TM	1RH5A	77.24	-20.07	16.4	144.91	24.04	26.53	64.39	-120.18	-28.2	86.19	16.06	27.7	18.49	174.31	59.13	36.94	49.81				
10TM	3K3FA	41.24	-20.86	38.86	-37.56	20.06	5.72	38.68	-139.05	26.03	-127.69	46.5	-31.87	22.96	-19.03	23.12	23.88	56.3				
10TM	3QKYA	-22.46	-11.33	-22.28	-40.15	-19.85	-51.85	-30.78	-50.11	-21.96	-27.63	-22.72	-14.84	-7.07	-39.87	-19.75	-35.46	-18.91				
10TM	4J72A	18.61	156.95	-7.15	129.76	-5.19	171.35	49.27	41.45	-12.68	-96.67	-32.79	111.81	-11.09	-72.89	-42.38	146.09	20.19				
10TM	1OTSA	34.62	-24.17	-6.37	-112.37	-38.91	-144.95	64.74	-128.53	77.84	7.94	44.38	2.47	-3.86	-103.97	-17.47	-168.71	60.03				
10TM	4WGV	-53.42	161.7	47.98	-61.37	3.59	-65.31	-21.23	-98.23	27.06	53.95	-41.86	-170.42	54.31	-115.29	44.44	116.29	-32.69				
11TM	4RP9A	11.83	-51.14	47.68	-44.33	21.54	-56.55	46.6	-42.9	35.45	-147.5	25.25	-62.47	71.74	-46.84	17.13	-28.79	38.98	53.51	-8.34		
11TM	4R0CA	35.81	93.43	18.15	159.48	30.41	-127.59	-29.62	46.24	10.23	165.76	31.73	83.34	48.48	-63.16	35.88	-156.98	17.6	101.97	1.45		
11TM	3B9YA	25.2	-29.82	30.12	-75.52	17.38	-33.34	37.43	-135.75	24.33	-120.26	38.07	-64.53	4.95	-59.54	21.94	-15.27	29.08	111.42	15.79		
11TM	4K1CA	-19.96	-116.17	10.94	-118.07	58.64	-104.09	-30.52	130.81	9.73	-122.57	32.02	-16.25	39.38	161.31	22.78	-128.18	-22.48	100.35	3.35		
11TM	1JB0A	-8.79	84.04	12.11	-167.84	-17.68	81.63	14.13	-155.93	-11.75	-66.27	-31.2	76.9	-27.15	-40.38	-20.95	-166.89	-27.84	83.47	-17.38		
12TM	4GC0A	26.59	-35.61	30.27	-36.05	-10.93	-81.1	19.69	-9.79	18.99	-91.31	27.34	7.41	21.76	-15.54	23.52	-19.99	-13.7	-99.65	16.59	3.87	8.93
12TM	3GIAA	33.35	43.8	-38.76	149.16	47.55	-55.92	23.19	-85.48	-23.4	-54.16	30.48	79.54	-32.77	-138.95	61.01	-29.89	63.64	154.45	-32.41	7.51	-11.22

12TM	3K07A	-16.86	-46.62	30.47	-57.58	22.98	62.96	2	-14.99	-10.57	66.02	25.57	-67.51	18.55	-82.97	42.16	-101.35	23.47	37.68	4.86	16.79	-29.11				
12TM	5DQQA	-20.09	-44.03	-23.4	128.64	-32.15	157.65	44.7	67.93	1.65	-154.04	28.24	121.13	-18.85	-23.75	-33.41	118.9	-1.93	40.56	56.76	64.44	4.24				
12TM	4KPPA	-38.72	-95.92	6.4	-64.12	19.93	-60.4	-23.4	109.21	-2.4	-139.9	48.35	13.76	40.19	-119.81	35.05	-88.09	-24	124.46	19.3	-124.59	17.91				
12TM	4ATVA	-0.22	-49.35	-9.66	-18.06	51.09	-110.08	-21.1	-147.24	-28.95	78.48	-22.11	-94.94	22.34	-107.88	-6.58	-33.72	-31.38	6.34	27.36	-118.07	-2.27				
12TM	4LZ6A	10.43	77.82	47.29	-76.33	4.92	-172.94	23.02	-50.25	15.57	159.9	19.99	15.59	20.56	78.13	19.5	-92.89	0.96	139.38	36.17	-52.3	30				
12TM	5KO2A	-16.03	-57.67	41.91	-114.79	-55.02	-155.91	0.47	-92.97	39	-67.9	6.39	117.9	-18.85	51.71	-13.83	-95.9	-2.85	138.97	-5.58	-59.67	8.97				
12TM	2DYRA	-20.42	144.53	-11.52	98.22	-11.16	-73.54	-28.43	-159.08	-15.36	108.61	17.52	155.03	-24.07	1.53	-15.26	-159.71	-20.29	141.39	-3.35	130.43	-18.63				
12TM	4C7RA	-53.05	-33.45	-44.94	-72.61	41.45	57.26	-57.69	-163.53	40.11	58.16	-34.56	72.1	-22.43	-75.24	57.44	36.64	-41	166.18	45.36	-90.99	42.22				
13TM	3RCEA	25.8	-54.61	16.11	-65.87	4.63	-173.65	-15.29	-53.36	10.51	-36.08	-10.25	159.34	-2.92	30.6	4.75	-91.39	3.42	16.78	-22.11	92.68	-42.43	156.51	25.3		
13TM	3S8GA	-22.73	159.89	-3.27	134.63	-16.6	-39.38	-38.78	-162.81	-10.85	139.36	22.91	-164.17	-23.31	9.56	-19.05	-169.47	-21.19	151.99	-10.41	-177.24	-20.51	13.87	-28.06		
13TM	4CZ8A	39.3	-165.46	-3	-94.18	13.8	-93.35	55.62	-80.78	-15.83	150.6	53.48	-33.18	29.89	-110.07	24.53	-90.4	-39.27	-58.31	22.21	-8.05	52.96	-96.24	-29.11		
13TM	4F35A	-6.94	148.56	20.3	145.09	-41.33	105.75	-2.9	60.14	16.76	-142.99	-12.06	67.63	24.74	-152.98	-14.35	91.84	36.16	-38.26	22.75	-89.63	3.91	95.74	39.33		
14TM	3QE7A	37.4	22.61	17.57	13.06	19.9	-18.86	-4.5	157.35	3.3	78.07	-14.15	-15.72	-33.43	-136.19	36.55	-17.47	48.56	-32.27	53.24	-16.64	-8.02	-124.3	-5.88	81.88	16.54
14TM	4IKVA	48.01	-40.35	33.85	-48.2	-19.89	-106.92	17.76	-10.62	26.4	-133.9	-1.1	36.57	38.93	178.88	-2.78	90.58	41.76	-51.28	31.41	-52.81	-16.53	-111.68	35.4	-12.53	11.16

Table S2: Converted signs of Ω and λ type dihedral angles for the given 103 proteins.

#TM	PDB	Ω1	λ1	Ω2	λ2	Ω3	λ3	Ω4	λ4	Ω5	λ5	Ω6	λ6	Ω7	λ7	Ω8	λ8	Ω9	λ9	Ω10	λ10	Ω11	λ11	Ω12	λ12	Ω13
3TM	2ZT9B	+	+	-																						
3TM	2BHWA	+	-	-																						
3TM	3ZE5A	-	+	-																						
3TM	4O9PA	+	-	-																						
3TM	1YQ3C	-	-	+																						
3TM	4X5MA	-	+	-																						
3TM	3RKOA	+	-	-																						
3TM	5AJIA	+	+	+																						
3TM	4U1WA	-	-	+																						
4TM	4HKRA	-	+	-	+	-																				
4TM	2BL2A	-	+	+	+	-																				
4TM	4YMKA	-	+	-	-	+																				
4TM	2UUHA	-	+	+	+	-																				
4TM	5TCXA	-	-	-	-	-																				
4TM	5ER7A	-	+	+	-	-																				
4TM	4WD8A	+	+	-	-	+																				
4TM	1KQFC	-	+	-	-	-																				
4TM	4RI2A	-	-	-	+	-																				
4TM	1Q90A	-	-	-	+	-																				
4TM	3EAMA	+	+	+	-	-																				
4TM	5DIRA	+	+	-	-	-																				
4TM	2ZUQA	-	-	-	-	-																				
5TM	4UC1A	+	+	-	+	-	-	-																		
5TM	3TUIA	+	-	+	+	+	+	+																		
5TM	4A2NB	-	+	-	+	-	-	+																		
5TM	1Q16C	+	-	+	+	+	+	+																		
5TM	3WU2A	-	-	-	+	-	+	-																		
5TM	3WVFA	+	-	+	-	+	+	-																		
5TM	3RGBC	-	-	-	+	-	-	-																		

5TM	4U9NA	+	+	+	-	+	+	+						
5TM	4NV5A	-	-	-	-	-	-	+						
6TM	3RGBB	+	-	+	-	+	+	+	+	+				
6TM	4MRSA	-	-	+	-	+	+	+	-	+				
6TM	3UX4A	-	-	-	+	-	-	-	-	-				
6TM	4B4AA	+	+	+	-	+	+	-	-	+				
6TM	3H90A	-	+	+	-	-	+	-	-	-				
6TM	5JWYA	+	-	+	-	+	-	-	+	+				
6TM	5I32A	+	-	+	-	+	+	-	-	+				
6TM	4P6VE	+	+	+	-	+	+	+	-	+				
6TM	3RVYA	-	-	-	-	-	+	+	+	-				
6TM	4O6MA	-	-	-	-	+	+	+	-	+				
6TM	2XOWA	+	+	+	-	+	+	-	+	-				
6TM	4XU4A	+	-	+	+	+	-	+	-	+				
6TM	1OKCA	+	-	+	+	-	-	+	+	+				
6TM	3RLBA	-	-	+	-	+	+	+	+	+				
6TM	4O6YA	-	-	-	-	-	+	-	-	+				
6TM	3WU2B	-	+	+	-	-	+	-	-	-				
6TM	3B4RB	+	+	+	-	-	+	+	-	-				
6TM	2R9RB	-	-	-	+	-	+	+	+	-				
7TM	2Z73A	-	-	-	+	+	+	+	-	-	-	-		
7TM	5SYTA	+	+	+	+	+	+	+	+	+	+	+		
7TM	4PGRA	+	-	-	-	+	+	+	+	-	-	-		
7TM	2DYRC	-	+	-	-	-	+	-	-	-	+	-		
7TM	5CTGA	-	+	-	+	-	+	+	-	-	+	-		
7TM	5AZBA	+	+	+	-	+	-	-	+	+	-	-		
7TM	5EGIA	+	-	+	+	-	-	+	-	+	+	+		
8TM	5DWYA	-	-	-	-	+	+	+	-	-	+	+	-	+
8TM	2VPZC	-	+	-	+	-	-	+	-	-	+	-	+	-
8TM	4QTNA	+	-	-	-	-	+	+	-	+	-	-	-	-
8TM	4J7CI	+	-	-	+	+	-	+	+	-	+	+	+	+
8TM	3RFUA	-	-	-	+	-	+	+	-	+	+	+	+	+
8TM	4P02A	-	+	-	-	-	+	-	+	+	-	+	+	-

**8TM**	**3TIJA**	+	+	+	-	+	+	+	-	+	-	+	+	+								
**9TM**	**4O9PB**	-	-	+	+	+	-	-	-	+	+	-	-	+	+	-						
**9TM**	**4TQ4A**	+	-	-	+	-	-	-	+	+	+	-	-	-	+	-						
**9TM**	**4Q2GA**	-	+	+	+	+	-	+	+	+	+	+	+	+	-	+						
**10TM**	**2ZXEA**	-	+	-	+	+	+	+	+	+	-	+	-	+	+	+	-	+				
**10TM**	**3QNQA**	+	+	+	-	+	-	+	-	+	-	-	-	-	+	+	+	+				
**10TM**	**4P6VB**	+	-	+	-	+	-	+	+	-	-	-	+	+	+	+	+	+				
**10TM**	**4QUVA**	+	-	+	-	+	-	+	-	-	+	-	+	-	+	-	-	+				
**10TM**	**5I20A**	+	-	+	+	+	+	+	-	+	+	+	-	+	+	+	-	-				
**10TM**	**2NQ2A**	+	+	-	+	+	-	+	-	-	+	-	-	+	-	+	-	+				
**10TM**	**3V5UA**	+	-	+	-	-	+	+	-	+	+	+	-	+	-	-	-	-				
**10TM**	**4WISA**	+	-	+	+	+	-	+	-	+	-	-	-	-	-	+	-	+				
**10TM**	**3M73A**	-	+	-	-	-	-	-	-	-	+	-	-	-	-	-	-	-				
**10TM**	**4N7WA**	+	-	+	-	+	+	+	-	+	+	+	-	+	-	+	-	-				
**10TM**	**1RH5A**	+	-	+	+	+	+	+	-	-	+	+	+	+	+	+	+	+				
**10TM**	**3K3FA**	+	-	+	-	+	+	+	-	+	-	+	-	+	-	+	+	+				
**10TM**	**3QKYA**	-	-	-	-	-	-	-	-	-	-	-	-	-	-	-	-	-				
**10TM**	**4J72A**	+	+	-	+	-	+	+	+	-	-	-	+	-	-	-	+	+				
**10TM**	**1OTSA**	+	-	-	-	-	-	+	-	+	+	+	+	-	-	-	-	+				
**10TM**	**4WGV**	-	+	+	-	+	-	-	-	+	+	-	-	+	-	+	+	-				
**11TM**	**4RP9A**	+	-	+	-	+	-	+	-	+	-	+	-	+	-	+	-	+	+	-		
**11TM**	**4R0CA**	+	+	+	+	+	-	-	+	+	+	+	+	+	-	+	-	+	+	+		
**11TM**	**3B9YA**	+	-	+	-	+	-	+	-	+	-	+	-	+	-	+	-	+	+	+		
**11TM**	**4K1CA**	-	-	+	-	+	-	-	+	+	-	+	-	+	+	+	-	-	+	+		
**11TM**	**1JB0A**	-	+	+	-	-	+	+	-	-	-	-	+	-	-	-	-	-	+	-		
**12TM**	**4GC0A**	+	-	+	-	-	-	+	-	+	-	+	+	+	-	+	-	-	-	+	+	+
**12TM**	**3GIAA**	+	+	-	+	+	-	+	-	-	-	+	+	-	-	+	-	+	+	-	+	-
**12TM**	**3K07A**	-	-	+	-	+	+	+	-	-	+	+	-	+	-	+	-	+	+	+	+	-
**12TM**	**5DQQA**	-	-	-	+	-	+	+	+	+	-	+	+	-	-	-	+	-	+	+	+	+
**12TM**	**4KPPA**	-	-	+	-	+	-	-	+	-	-	+	+	+	-	+	-	-	+	+	-	+
**12TM**	**4ATVA**	-	-	-	-	+	-	-	-	-	+	-	-	+	-	-	-	-	+	+	-	-
**12TM**	**4LZ6A**	+	+	+	-	+	-	+	-	+	+	+	+	+	+	+	-	+	+	+	-	+
**12TM**	**5KO2A**	-	-	+	-	-	-	+	-	+	-	+	+	-	+	-	-	-	+	-	-	+

**12TM**	**2DYRA**	-	+	-	+	-	-	-	-	-	+	+	+	-	+	-	-	-	+	-	+	-				
**12TM**	**4C7RA**	-	-	-	-	+	+	-	-	+	+	-	+	-	-	+	+	-	+	+	-	+				
**13TM**	**3RCEA**	+	-	+	-	+	-	-	-	+	-	-	+	-	+	+	-	+	+	-	+	-	+	+		
**13TM**	**3S8GA**	-	+	-	+	-	-	-	-	-	+	+	-	-	+	-	-	-	+	-	-	-	+	-		
**13TM**	**4CZ8A**	+	-	-	-	+	-	+	-	-	+	+	-	+	-	+	-	-	-	+	-	+	-	-		
**13TM**	**4F35A**	-	+	+	+	-	+	-	+	+	-	-	+	+	-	-	+	+	-	+	-	+	+	+		
**14TM**	**3QE7A**	+	+	+	+	+	-	-	+	+	+	-	-	-	-	+	-	+	-	+	-	-	-	-	+	+
**14TM**	**4IKVA**	+	-	+	-	-	-	+	-	+	-	-	+	+	+	-	+	+	-	+	-	-	-	+	-	+

Table S3: TM protein pairs showing symmetric macroscopic structure

TM (# of proteins in the dataset)	Configuration of λ angle sign	PDB IDs
**3TM (9)**	(+)	3ZE5A, 5AJIA, 4X5MA, 5AJIA
	(-)	4O9PA, 1YQ3C, 3RKOA, 2BHWA, 4U1WA
**4TM (13)**	(+, -)	4WD8A, 5DIRA, 4YMKA, 5ER7A, 1KQFC, 3EAMA
	(-, +)	1Q90A
	(+,+)	4HKRA, 2BL2, 2UUHA
	(-, -)	5TCXA, 2ZUQA
**5TM (9)**	(+, +, -)	4UC1A, 4A2NB
	(-, -, +)	3WVFA
	(-, +, -)	3RGBC
	(+, -, +)	4U9NA
**6TM (18)**	(-, +, -, -)	3UX4A, 4XU4A
	(+, -, +, +)	2XOWA
	(+, -, +, -)	4B4AA, 3H90A, 4P6VE, 3WU2B, 3B4RB
	(-, +, -, +)	1OKCA

Table S4: TM protein pairs showing symmetric macroscopic structure

TM (# of proteins in the dataset)	Configuration of λ angle sign	PDB IDs
**3TM (9)**	**(+)**	3ZE5A, 5AJIA
	**(-)**	4O9PA, 1YQ3C, 3RKOA
**4TM (13)**	**(+, -)**	4WD8A, 5DIRA
	**(-, +)**	1Q90A
**5TM (9)**	**(+, +, -)**	4A2NB
	**(-, -, +)**	3WVFA

Here, PDB codes of 103 non-homologous helical membrane proteins are given in the first column and dihedral angles for all consecutive Ω and λ types in given in the subsequent columns.

Table S5: All 103 PDB IDs, Protein names and their classification used in this work.

#	PDB ID	Protein Name	Classification
**1**	2ZT9B	Cytochrome b6f	Photosynthesis
**2**	2BHWA	Pea Light-Harvesting	Photosynthesis
**3**	3ZE5A	Diacylglycerol kinase	Transferase
**4**	4O9PA	NAD(P) transhydrogenase	Transhydrogenase
**5**	1YQ3C	Succinate dehydrogenase	Oxidoreductase
**6**	4X5MA	SemiSWEET	Transport Protein
**7**	3RKOA	Respiratory complex I	Oxidoreductase
**8**	5AJIA	Mechanosensitive channel	Transport Protein
**9**	4U1WA	Glutamate receptor 2	Transport Protein
**10**	4HKRA	Calcium release-activated calcium (CRAC) channel	Transport Protein
**11**	2BL2A	V-type Sodium ATPase	Hydrolase
**12**	4YMKA	Acyl-CoA desaturase	Oxidoreductase
**13**	2UUHA	Leukotriene C4 Synthase	Lyase
**14**	5TCXA	Tetraspanin	Cell Invasion
**15**	5ER7A	Connexin	Calcium Binding Protein
**16**	4WD8A	Bestrophin	Membrane Protein
**17**	1KQFC	Formate dehydrogenase	Oxidoreductase
**18**	4RI2A	Photoprotective protein PsbS	Membrane Protein
**19**	1Q90A	Cytochrome b6f	Photosynthesis
**20**	3EAMA	Proton-gated ion channel	Transport Protein
**21**	5DIRA	Lipoprotein signal	Hydrolase
**22**	2ZUQA	Disulfide bond formation protein	Oxidoreductase / Immune System
**23**	4UC1A	Translocator protein (TSPO)	Membrane Protein

**24**	3TUIA	ABC transporter	Hydrolase/ Transport Protein
**25**	4A2NB	Integral Membrane Methyltransferase	Transferase
**26**	1Q16C	Respiratory Nitrate Reductase	Oxidoreductase
**27**	3WU2A	Photosystem II	Photosynthesis
**28**	3WVFA	Bacterial YidC	Chaperone
**29**	3RGBC	Methane monooxygenase	Oxidoreductase
**30**	4U9NA	Magnesium ion transporter-E	Metal Transport
**31**	4NV5A	Vitamin K epoxide reductase	Oxidoreductase
**32**	3RGBB	Methane monooxygenase	Oxidoreductase
**33**	4MRSA	ABC transporter	Transport Protein
**34**	3UX4A	Acid-activated urea channel	Transport Protein
**35**	4B4AA	Twin-Arginine Protein	Transport Protein
**36**	3H90A	Zinc transporter	Transport Protein
**37**	5JWYA	Lipid phosphate phosphatase	Hydrolase
**38**	5I32A	Aquaporin’s	Membrane Protein/Water Transport
**39**	4P6VE	NADH-quinone reductase	Oxidoreductase
**40**	3RVYA	Voltage-gated sodium (Na(V)) channel	Metal Transport
**41**	4O6MA	CDP-alcohol phosphotransferase	Transferase
**42**	2XOWA	Rhomboid Protease	Hydrolase
**43**	4XU4A	Insig-related proteins	Membrane Protein
**44**	1OKCA	Mitochondrial ADP/ATP Carrier	Transport Protein
**45**	3RLBA	Energy coupling factor (ECF) transporters	Thiamine Binding Protein
**46**	4O6YA	Ascorbate-dependent oxidoreductase	Oxidoreductase
**47**	3WU2B	Photosystem II	Photosynthesis
**48**	3B4RB	Site-2 protease	Hydrolase

**49**	2R9B	Voltage-dependent K+ (Kv) channel	Transport Protein
**50**	2Z73A	Squid rhodopsin	Membrane Protein GPCR
**51**	5SYTA	CaaX Protease	Hydrolase
**52**	4PGRA	Protein YetJ	Membrane Protein
**53**	2DYRC	Cytochrome c oxidase	Oxidoreductase
**54**	5CTGA	SWEET transporter	Transport Protein
**55**	5AZBA	Prolipoprotein diacylglyceryl transferase	Transferase
**56**	5EGIA	Trimeric Intracellular Cation channel	Transport Protein
**57**	5DWYA	Glutamate transporter	Transport Protein
**58**	2VPZC	Bacterial polysulfide reductase	Oxidoreductase
**59**	4QTNA	Vitamin B3 transporter	Transport Protein
**60**	4J7CI	KtrAB potassium transporter	Transport Protein
**61**	3RFUA	PIB-ATPase	Hydrolase
**62**	4P02A	Cellulose synthase	Transferase
**63**	3TIJA	Concentrative nucleoside transporter	Transport Protein
**64**	4O9PB	NAD(P) transhydrogenase	Transhydrogenase
**65**	4TQ4	Prenyltransferases	Transferase
**66**	4Q2GA	CDP-DAG synthetase	Transferase
**67**	2ZXEA	Sodium-potassium ATPase	Hydrolase/ Transport Protein
**68**	3QNQA	Saccharide transporter	Transport Protein
**69**	4P6VB	Ubiquinone oxidoreductase	Oxidoreductase
**70**	4QUVA	Sterol reductase	Oxidoreductase
**71**	5I20A	Drug/metabolite transporter	Transport Protein
**72**	2NQ2A	ABC transporter	Metal Transport
**73**	3V5UA	Sodium/Calcium Exchanger	Metal Transport

**74**	4WISA	TMEM16 lipid scramblase	Lipid Transport
**75**	3M73A	Tellurite-resistance/Dicarboxylate transporter	Transport Protein
**76**	4N7WA	Bile Acid:Na+ symporter (BASS) transporter	Transport Protein
**77**	1RH5A	Protein-conducting channel	Transport Protein
**78**	3K3FA	Urea Transporter	Transport Protein
**79**	3QKYA	BamD	Membrane Protein
**80**	4J72A	Phospho-MurNAc-Pentapeptide translocase	Transferase
**81**	4RP9A	Vitamin C transporter	Transport Protein
**82**	4R0CA	YdaH transporter	Transport Protein
**83**	3B9YA	Ammonia and urea transporters	Transport Protein
**84**	4K1CA	Calcium/Proton Exchanger	Metal Transport
**85**	1JB0A	Cyanobacterial Photosystem I	Photosynthesis
**86**	4WGVA	Divalent metal ion transporter (DMT)	Transport Protein
**87**	4GC0A	Proton: xylose symporter XylE	Transport Protein
**88**	3GIAA	ApcT Transporter	Transport Protein
**89**	3K07A	Efflux transporter CusA	Transport Protein
**90**	5DQQA	Two-pore channel TPC1	Transport Protein
**91**	4KPPA	Ca(2+)/cation antiporter	Transport Protein
**92**	4ATVA	Sodium-Proton Antiporter	Transport Protein
**93**	4LZ6A	Multidrug & toxic compound extrusion transporter	Transport Protein
**94**	5KO2A	Multidrug Transporter P-glycoprotein	Hydrolase
**95**	2DYRA	Cytochrome c oxidase	Oxidoreductase
**96**	3RCEA	Oligosaccharyltransferase	Transferase
**97**	3S8GA	Cytochrome c oxidase	Oxidoreductase
**98**	4CZ8A	Sodium/proton antiporter	Transport Protein

**99**	4C7RA	Trimeric betaine transporter BetP	Transport Protein
**100**	3QE7A	Uracil Transporter-UraA	Transport Protein
**101**	4IKVA	Peptide transporter POT	Transport Protein
**102**	1OTSA	ClC Chloride channel	Transport Protein
**103**	4F35A	Dicarboxylate/sodium symporter	Transport Protein
